# Reactive oxygen species/glutathione dual sensitive nanoparticles with encapsulation of miR155 and curcumin for synergized cancer immunotherapy

**DOI:** 10.1186/s12951-024-02575-5

**Published:** 2024-07-08

**Authors:** Kangkang Li, Juan Wang, Yi Xie, Ziyao Lu, Wen Sun, Kaixuan Wang, Jinxin Liang, Xuehong Chen

**Affiliations:** 1 School of Basic Medicine, Qingdao University, Qingdao, China; 2 Pharmacy Department, Qingdao Hospital, University of Health and Rehabilitation Sciences (Qingdao Municipal Hospital), Qingdao, China; 3 Department of Neurosurgery, Affiliated Hospital of Qingdao University, Qingdao, China

**Keywords:** Curcumin, miR155, Immunotherapy, Melanoma, Triple negative breast cancer

## Abstract

Considerable attention has been directed towards exploring the potential efficacy of miR-155 in the realm of cancer immunotherapy. Elevated levels of miR-155 in dendritic cells (DCs) have been shown to enhance their maturation, migration, cytokine secretion, and their ability to promote T cell activation. In addition, overexpression of mir155 in M2 macrophages boost the polarization towards the M1 phenotype. Conversely, miR-155 has the propensity to induce the accumulation of immunosuppressive cells like regulatory T cells (Tregs) and myeloid-derived suppressor cells (MDSCs) in the tumor tissue. To account for this discrepancy, it is imperative to get help from a drug that could deal with immunosuppressive effect. Curcumin (CUR) exhibits the capacity to prompt Tregs converse into T helper 1 cells, fostering the polarization of M2 tumor-associated macrophage towards the M1 phenotype, and impeding the recruitment and aggregation of MDSCs within the tumor microenvironment. Nonetheless, CUR is known to exert an immunosuppressive impact on DCs by hindering the expression of maturation markers, cytokines, and chemokines, thereby prevent DCs response to immunostimulatory agents. Hence, a reactive oxygen species/glutathione dual responsive drug conveyance platform (CUR/miR155@DssD-Hb NPs) was devised to co-deliver CUR and miR155, with the aim of exploring their synergistic potential in bolstering a sustained and robust anti-tumor immune response. In vitro and in vivo results have suggested that CUR/miR155@DssD-Hb NPs can effectively inhibit the viability of 4T1 and B16F10 tumor cells, trigger the release of damage associated molecular patterns, stimulate DCs maturation, subsequent activation of CD8^+^ T cells, diminish immunosuppressive cell populations (MDSCs, Tregs, M2 TAMs and exhausted T cells), promote the formation of long-term immunity and lessen the formation of metastatic nodules in the lungs. In summary, the co-delivery system integrating CUR and miR155 (CUR/miR155@DssD-Hb NPs) demonstrates promise as a promising strategy for the immunotherapy of melanoma and triple negative breast cancer.

**Graphical abstract:**

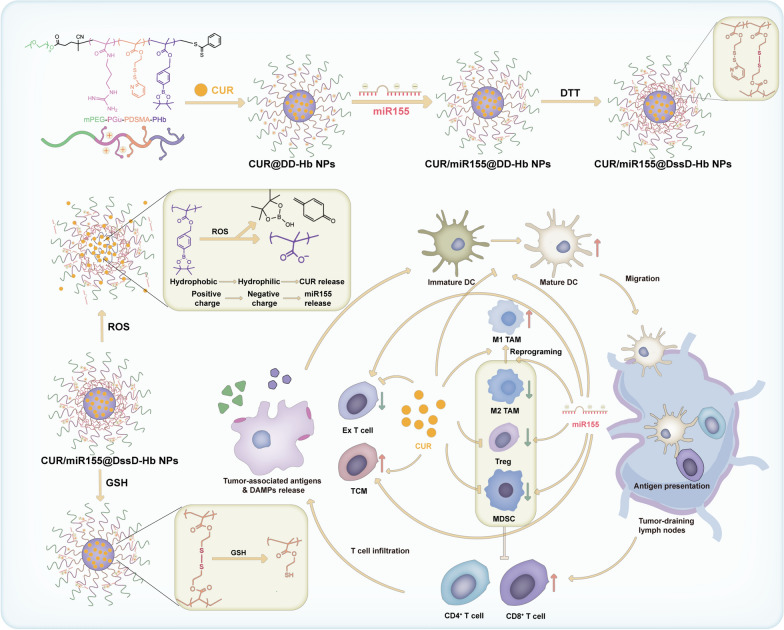

**Supplementary Information:**

The online version contains supplementary material available at 10.1186/s12951-024-02575-5.

## Introduction

MicroRNAs (miRNA) are evolutionarily conserved small non-coding RNAs molecules that post transcriptionally modulate the expression of multiple target genes and are hence implicated in a wide series of cellular and developmental processes [1]. In recent years, miRNAs have emerged as novel targets for cancer therapy. A growing number of evidences from clinical and preclinical studies have highlighted that dysregulation of miRNA function contributes to the progression of cancer, consequently miRNA can be an effective target in therapy.

miR-155 is considered an oncogenic miRNA, connecting inflammation with tumorigenesis in certain cancer types [2]. Several studies have revealed that inhibiting miR-155 in vitro enhances apoptosis, and reduced proliferation, migration, and colony formation in diverse cancers such as osteosarcoma, multiple myeloma, glioblastoma multiforme, and endometrial carcinoma [3–5]. However, conflicting reports have indicated that elevated levels of miR-155 in tumors correlate with enhanced overall survival in patients with breast cancer, colon cancer, and melanoma [6–9], suggesting a dual role for miR-155 that may stem from its pivotal involvement in innate and adaptive immune responses [8, 10, 11]. For example, the higher miR-155 levels in breast tumors are associated with favorable antitumor immune infiltration and improved patient outcomes. The elevated miR155 level  within breast cancer cells has been shown to suppress tumor progression by augmenting the recruitment of anti-tumor immune cells [12, 13]. Additionally, increased miR-155 levels in various immune cell populations, including CD4^+^ and CD8^+^ T cell, M1 tumor-associated macrophages (TAMs), dendritic cells (DCs), and natural killer cells, can enhance their anti-tumor activity. Elevated miR-155 expression in TAMs is linked to the conversion of pro-tumoral M2 TAMs into the anti-tumor M1 subtype [12, 14, 15]. Gao et al. designed an erythrocyte membrane-coated nucleic acid nanogel to deliver therapeutic miR155, which demonstrated the ability to reprogram microglia and macrophages from a pro-invasive M2 phenotype to an anti-tumor M1 phenotype [16]. Notably, studies have demonstrated that the absence of miR-155 in DCs compromises their maturation, migration, cytokine production, and their capacity to activate T cells. [17]. In addition, recent research underscores that T cell-specific miR-155 expression is necessary for optimal antitumor immunity in various experimental models [18–21]. However, miR-155 expression in immune cells cannot be considered exclusively oncogenic or tumor suppressive, in fact its role is immune cell-type dependent. miR-155 is a direct target of an important transcription factor that is required for the correct and functional development of regulatory T cells (Tregs), fork head box P3 (FOXP3) [22, 23]. On the other hand, the expression of miR-155 in myeloid-derived suppressor cells (MDSCs) is necessary for the recruitment of MDSCs in the malignant site and for promoting tumor growth [22]. In the last years, while several miR155 preparations for cancer immunotherapy have been developed with very promising results, these delivery systems are mainly focused on the targeted delivery to TAMs and DCs [16, 24]. It is challenging to achieve the desired therapeutic effect solely through miR155.

To account for this discrepancy, it is imperative to get help from a drug that could play pivotal roles not only in killing tumor cells but also in dealing with the immune environment such as reducing immunosuppressive factors. Cancer immunotherapy utilizing plant-derived natural compounds has emerged as an innovative therapeutic field, with various phytochemicals exhibiting immunomodulatory properties by modulating diverse immune signaling pathways [1]. Among these natural agents, curcumin (CUR) has garnered significant interest for its potential in cancer immunotherapy. Some studies have indicated that this phytochemical could target Tregs and convert them into Th1, shift M2 TAMs towards the anti-tumorigenic M1 phenotype, and impede the recruitment and accumulation of MDSCs in the tumor microenvironment (TME) [25, 26]. In addition, CUR impacts various growth factor receptors, cell adhesion molecules, and cellular processes involved in tumor growth, metastasis, apoptosis, and multidrug resistance [27]. Nonetheless, curcumin's immunosuppressive impact on DCs, characterized by the inhibition of maturation markers, cytokines, and chemokine expression, could deter the response of DCs to immunostimulatory agents [26]. Given that the application of CUR is accompanied by an immunosuppressant effect on DCs while miR155 acts as a facilitator for DCs maturation, we expected that miR155 could potentially counteract the negative repercussions induced by curcumin. Furthermore, curcumin might be able to directly kill tumor cells and inhibit the recruitment and accumulation of MDSCs and Tregs in TME, thereby resulting in the minimization of the negative effects induced by miR155.

Here, we constructed a reactive oxygen species/glutathione (ROS/GSH) dual sensitive drug delivery system (CUR/miR155@DssD-Hb NPs) to co-delivery CUR and miR155 and investigated whether CUR and miR155 can complement each other for activating the long-lasting anti-tumor immune response. PHb serving as hydrophobic segments forms the ROS-responsive core for self-assembling into nanoparticles and loading of lipophilic CUR. GSH-sensitive PDSMA facilitates the crosslinking of themselves via disulfide bonds to improve stability and avoid the undesirable leakage during circulation. Negatively charged miR155 is loaded onto PGu through electrostatic interactions. Upon reaching the TME, the distinct tumor ROS environment triggers the transformation from hydrophobic borate to hydrophilic carboxyl groups, facilitating the controlled release of curcumin. Finally, our findings illustrated that these nanocomplexes effectively enhanced anti-tumor immune responses and reshaped the immunosuppressive TME, leading to notable tumor regression and reduced pulmonary metastatic nodules. Our results underscored the potential of co-delivering curcumin and miR155 as a promising approach for melanoma and triple negative breast cancer (TNBC) immunotherapy.

## Materials and methods

### Materials

All the chemical agents were purchased from Macklin (Shanghai, China). Confocal dish, 96-well plates, 6-well plates cell culture flasks and centrifuge tubes were purchased from SAINING life science (China). miRNA-NC, miR155 and Cy5-miRNA was purchased from GenePharma (Shanghai, China). CUR was purchased from Macklin (Shanghai, China). Lipo3000, 2′,7′-Dichlorodihydrofluorescein diacetate (DCFH-DA), MTT solution, 2% phosphotungstic acid, ATP Content Assay Kit and Lyso-Tracker Green was purchased from Solarbio Life Sciences (China). Immunohistochemistry (IHC) detection system kit and antibodies were purchased from Bioss (Beijing, China). Anti-rabbit calreticulin polyclonal antibody and antirabbit CoraLite647@ secondary antibody were purchased from Proteintech Group (USA). Anti-CD80-FITC, anti-CD11c-APC, anti-Gr-1-APC, anti-F4/80-APC, Anti-CD8-APC, Anti-CD4-FITC, Anti-CD3-APC, Anti-CD25-APC, Anti-FoxP3-PE, Anti-CD11b-PE, Anti-Ly6G-FITC were purchased from MULTISCIENCES (Hangzhou, China). Anti-TCF-1-PE, Anti-IFN-γ-PE, Anti-CD44-FITC and Anti-CD62L-APC were purchased from BioLegend (USA). Anti-CD206-PE (CAT.No: FMP206-01–100), Anti-CD86-PE (CAT.No: FMP086-02–100) and anti-CD8-PE (CAT.No: FMP008-02–100) were purchased from 4A Biotech (Suzhou, China). Bouin's fixative solution, 4% paraformaldehyde fixative solution, crystal violet staining solution (0.1%), mouse IFN-γ ELISA KIT, mouse IL-10 ELISA KIT, mouse IL-12 p70 ELISA KIT, mouse HMGB1 ELISA KIT, mouse Recombinant GM-CSF, HMGB1 Mouse mAb, Ki67 (9A9) Mouse mAb, PD-L1 Mouse mAb, Calreticulin Mouse mAb, DAPI staining solution, Goat Anti-Mouse IgG (H + L) Alexa Fluor 488, FITC-Annexin V/PI Apoptosis Detection Kit, Serum-Free Cell Freezing Medium, RPM1640 cell culture medium, DMEM cell culture medium, Penicillin–Streptomycin Solution, Cell Cycle and Apoptosis Analysis Kit were purchased from Share-bio, Shang Hai (Shanghai, China). Fetal Bovine Serum (FBS, FBS BS-1101) were purchased from Inner Mongolia Opcel Biotechnology Co.,Ltd (China).

### Mice and cell line

Male C57 mice and female Balb/c mice with body weight of 18–22 g were purchased from Pengyue (Jinan, China). The mice were kept under specific pathogen-free conditions at 23 ± 2 °C with water and food given ad libitum. The experiments are carried out in accordance with the protocol approved by the Animal Research Committee of Qingdao University.

HEK-293, B16F10 and 4T1 cells were obtained from Share-bio, Shang Hai (Shanghai, China) and cultured in RPM1640 and DMEM supplemented with 10% FBS at 37 ℃ in a humidified atmosphere with 5% CO_2_.

### Preparation and characterization of nanoparticles

#### Preparation of nanoparticles

The preparation of CUR@DD-Hb NPs involved the thin-film hydration method. Initially, 10 mg of mPEG-PGu-PDMSA-PHb was dissolved in 4 mL of acetonitrile. Subsequently, 1 mL of methanol containing 1 mg of CUR was added to the aforementioned solution. The solvent was removed using a rotary evaporator, resulting in a thin film that was hydrated with 2 mL of PBS solution at 60 °C for 30 min to yield a micellar solution. Following centrifugation at 3000 rpm for 5 min and filtration through a 0.22 μm filter, any unencapsulated CUR was eliminated, yielding CUR@DD-Hb NPs. For the formulation of miR155 nanocomplexes, CUR@DD-Hb NPs were incubated with miRNA-NC, Cy5-miRNA, and miR155 at varying N/P molar ratios at room temperature for 30 min to produce CUR/miRNA-NC@DD-Hb NPs, CUR/Cy5-miRNA@DD-Hb NPs, and CUR/miR155@DD-Hb NPs.

To generate cross-linked CUR/miR155@DssD-Hb NPs, non-cross-linked CUR/miR155@DD-Hb NPs (5 mg) were dispersed in 10 mL of PBS, and 1,4-dithiothreitol (DTT) (10 mM) solution were added to the solution [28]. The mixture was stirred for 24 h at room temperature. Subsequently, the cross-linked NPs were collected by dialysis, and characterized via transmission electron microscopy (TEM) and zeta potential measurements.

#### Particle size, zeta potential and morphology measurements

Particle size and zeta potential of the nanocomplexes were assessed using a Malvern Nano-ZS device (Malvern, UK). All findings presented are the average of three experimental trials. The morphology was examined through JEM-2100 TEM (JEOL, Tokyo, Japan).

#### Drug loading efficiency (LE) and encapsulation efficiency (EE) of CUR

The determination of LE and EE of CUR utilized an HPLC method. In summary, nanocomplexes were dispersed in methanol, and the concentration of CUR was analyzed using the Acquity Hclass plus HPLC system (Waters, Singapore). LE and EE of CUR were computed using the following equations:

$$LE\left(\%\right)=\frac{{W}_{loaded CUR}}{{W}_{Nanocomplexes}} \times 100\%$$

$$EE\left(\%\right)=\frac{{W}_{loaded CUR}}{{W}_{total CUR}} \times 100\%$$

where ${W}_{loaded CUR}$, ${W}_{total CUR}$ and ${W}_{Nanocomplexes}$ represent the weight of loaded and total CUR, nanocomplexes, respectively.

Ultrafiltration method was used to determine the LE and EE of Cy5-miRNA. CUR/Cy5-miRNA@DssD-Hb NPs were added into centrifugal filter devices (50 kDa, Millipore, US) and centrifuged at 4000 rpm for 10 min. The uncomplexed Cy5-miRNA was collected and quantified by SynergyMx microplate reader (BioTek, US) (Ex: 640 nm; Em: 660 nm). The EE of Cy5-miRNA was calculated by the following equations:

$$LE\left(\%\right)=\frac{{W}_{loaded Cy5-miRNA}}{{W}_{Nanocomplexes}} \times 100\%$$

$$EE\left(\%\right)=\frac{{W}_{loaded Cy5-miRNA}}{{W}_{total Cy5-miRNA} } \times 100\%$$

where ${W}_{loaded Cy5-miRNA}$, ${W}_{total Cy5-miRNA}$ and ${W}_{Nanocomplexes}$ represent the weight of loaded and total Cy5-miRNA, nanocomplexes, respectively.

### In vitro drug release and nanocomplexes stability assay

The release profiles of CUR/miR155@DssD-Hb NPs responsive to both ROS and GSH were explored via a dialysis methodology. Specifically, various CUR formulations were enclosed within dialysis bags (3500 Da) and co-cultured with 50 mL PBS buffer (0.01 M, pH = 7.4) alongside either 1 mM H_2_O_2_ or 10 mM GSH, or without, in a shaker incubator set at 37 °C. At designated time points, 1 mL of the release medium was withdrawn and replaced with fresh medium to sustain the volume. The concentration of CUR was quantified through HPLC analysis. The release of Cy5-miRNA was determined via Ultrafiltration method. Formulations were co-cultured with PBS buffer (0.01 M, pH = 7.4) alongside either 1 mM H_2_O_2_ or 10 mM GSH, or without. At designated time points, centrifugal filter devices (50 kDa, Millipore, US) were used to obtain free miR155.

As for stability assessment, the nanoparticles were exposed to conditions with or without 10 mM GSH and 10% FBS. Their particle sizes were investigated using dynamic light scattering (DLS) as previously described.

### ROS and GSH responsiveness evaluation

To investigate the ROS and GSH responsiveness, CUR/miR155@DssD-Hb NPs was incubated with GSH and H_2_O_2_ for 2 h. Subsequently, the particle sizes were investigated via the DLS technique as mentioned above. Additionally, the morphological changes of nanoparticles treated with GSH and H_2_O_2_ were observed and captured by TEM.

### Cellular uptake assay

To evaluate cellular uptake efficiency and distribution, Cy5-labeled miRNA was employed in 4T1 and B16F10 cells through flow cytometry and confocal laser scanning microscopy (CLSM). For flow cytometry analysis, tumor cells were plated in 12-well dishes at a concentration of 10^5^ cells per well. FBS-free culture medium containing CUR/Cy5-miRNA@DssD-Hb NPs and Cy5-miRNA@lipo was administered to the tumor cells for 1 and 4 h. Subsequently, the tumor cells underwent a cold PBS (pH 7.4) wash, trypsinization, and harvest, culminating in evaluation through CytoFlex S flow cytometry (Beckman, Villepinte, France). For CLSM, tumor cells were cultured on glass coverslips within a 12-well plate, then treated with CUR/Cy5-miRNA@DssD-Hb NPs and free Cy5-miRNA for 1 h and 4 h. Following incubation, the cells were sequentially subjected to Lyso-Tracker Green, PBS (pH 7.4), 4% formaldehyde, and DAPI. Subsequent observation was conducted utilizing the STELLARIS 5 confocal microscope (Leica, Germany).

### In vitro cell cytotoxicity

The MTT assay was implemented for the assessment of in vitro cytotoxicity of the formulations. Specifically, 5 × 10^4^ cells were seeded into 96-well plates and exposed to growth medium devoid of FBS containing the formulations, followed by a 24 h incubation period. Subsequently, MTT reagent was introduced to the plates and allowed to incubate for 4 h, after which DMSO was applied to dissolve the formazan crystals. The optical density at 490 nm was quantified using a SynergyMx microplate reader (BioTek, US). Cell viability was then computed utilizing the subsequent equations:

$$Cell viability\left(\%\right)=\frac{{A}_{Treatment}{-A}_{Blank}}{{A}_{Control}{-A}_{Blank}} \times 100\%$$

where A_Treatment_, A_Control_ and A_Blank_ denote the absorbance of treatment group, control group and PBS group.

### In vitro apoptosis and cell cycle analysis

Apoptosis was explored through Annexin V-FITC-PI staining followed by flow cytometry analysis. Specifically, 4T1 and B16F10 cells subjected to nanoformulations (CUR: 5 μg/mL) for 24 h were trypsinized, collected, and then stained with PI and Annexin V-FITC according to the manufacturer’s instructions. Subsequently, analysis was conducted utilizing a CytoFlex S flow cytometer (Beckman, California, USA). For cell cycle analysis, 4T1 and B16F10 cells treated with nanoformulations for 24 h underwent trypsinization, harvesting, and fixation in 70% ice-cold ethanol at 4 °C over a period of 12 h. Following PI staining for 30 min, the distribution of the cell cycle was determined using a CytoFlex S flow cytometer (Beckman, California, USA).

### In vitro ROS production

In vitro detection of ROS levels was assessed utilizing flow cytometry and CLSM. For the flow cytometry analysis, 4T1 and B16F10 cells exposed to nanoformulations with a concentration of 5 μg/mL CUR for 24 h were subjected to staining with DCFH-DA for 30 min, fixed with 4% paraformaldehyde, and examined using the CytoFlex S flow cytometer (Beckman, California, USA). For CLSM observation, tumor cells were cultured in confocal dishes and treated with nanoformulations at a concentration of 5 μg/mL CUR for 24 h. Following this, the tumor cells were exposed to fresh medium containing 10 µg/mL DCFH-DA for 30 min, fixed with 4% paraformaldehyde, and observed using the STELLARIS 5 confocal microscope (Leica, Germany).

### Wound-healing and transwell assay

B16F10 and 4T1 tumor cells were cultured in 12-well plates at a density of 3 × 10^6^ cells per well. Upon reaching a confluent monolayer, a 200 μL pipette tip was utilized to create a scratch. Subsequently, the cells were washed twice with PBS, followed by the addition of a culture medium containing various formulations (CUR: 1 μg/mL) without FBS. After 48 h of incubation, images were captured using an inverted microscope, the Eclipse Ts2 (Nikon, Tokyo, Japan).

Transwell inserts were coated with a 100 μL Matrigel solution (0.2–0.3 mg/mL, diluted with serum-free medium) and left to incubate overnight at 37 ℃. Subsequently, fresh medium containing 4 × 10^4^ cells and different formulations (CUR: 1 μg/mL) were added to the inserts, which were then positioned in 24-well plates containing 0.6 mL of medium supplemented with 15% FBS. Following 48 h of incubation, the inserts were washed, fixed, and stained with 0.1% crystal violet. Images were captured using an inverted microscope, the Eclipse Ts2 (Nikon, Tokyo, Japan).

### In vitro ICD induction assay

#### Calreticulin (CRT) externalization

The externalization of CRT was explored through both flow cytometry and CLSM. Briefly, following a 24 h treatment with nanoformulations, 4T1 and B16F10 cells were trypsinized, collected, and then stained with CRT antibodies at 4 °C overnight, along with fluorescence-labeled secondary antibodies. The samples were then analyzed using the CytoFlex S flow cytometer (Beckman, California, USA). For CLSM examination, tumor cells were cultured in a 12-well plate containing glass coverslips and subjected to staining with CRT antibodies, fluorescence-labeled secondary antibodies, and DAPI. Subsequently, CRT externalization was observed using the STELLARIS 5 confocal microscope (Leica, Germany).

#### High mobility group protein 1 (HMGB1) and adenosine triphosphate (ATP) release assay

After incubation with formulations for 24 h, the culture medium was collected and analyzed using ELISA Kits in accordance with manufactures’ instructions. The fluorescence intensity was measured by a SynergyMx microplate reader (BioTek, US).

### Maturation analysis of bone-marrow-derived dendritic cells (BMDCs)

BMDCs derived from six-week-old female BALB/c and male C57 mice were employed to assess the capacity of CUR/miR155@DssD-Hb NPs to stimulate and activate DCs. GM-CSF was administered to facilitate the differentiation of BMDCs. B16F10 and 4T1 cells treated with the formulation were then cocultured with immature BMDCs for a duration of 24 h. Following this incubation period, antibodies (CD11c, CD80, CD86) were utilized in accordance with the manufacturers' instructions, and the maturation of DCs was examined using the CytoFlex S flow cytometer (Beckman, California, USA).

### In vivo biodistribution in 4T1 tumor mice

Mice bearing 4T1 tumors were divided into random groups and administered Cy5-labeled nanocomplexes via tail vein injection. Fluorescence signals were assessed at specified time points using the IVIS Lumina XRMS III Image System (PerkinElmer, US). After 48 h, the mice were euthanized, and the major organs along with the tumor were excised and subjected to imaging.

### In vivo antitumor efficacy in 4T1 tumor mice

On 7th tumor inoculation, the mice were randomly divided into four groups and received the formulation via tail vein injection. The grouping and doses for the mice were as follows: (1) PBS; (2) Free CUR; (3) CUR@DssD-Hb NPs; (4) CUR/miR155@DssD-Hb NPs (CUR: 10 mg/kg, N/P = 10). Subsequently, CUR formulations were administrated every two days and all mice were given five injections. Throughout this period, the mice's body weight and tumor dimensions were meticulously documented. On the 17th day, the mice were euthanized, and the tumor tissues were extracted, weighed, and preserved in 4% paraformaldehyde for subsequent investigations. The tumor volume and the tumor growth inhibition ratio (TGI) were then computed utilizing the ensuing equations:

$$Tumor volume ({mm}^{3})=\frac{a\times {b}^{2}}{2}$$

where a and b denote the longest and shortest diameters of tumor.

$$TGI \left(\%\right)=(1-\frac{{(V}_{0}/{V}_{T})tested group}{{(V}_{0}/{V}_{T})PBS group})\times 100$$

where V_t_ and V_0_ denote the tumor volume at the beginning and ending, respectively.

### In vivo immune responses

For the examination of immune cell components within the tumor tissues, on the 13th day, tumor tissues, TDLNs, and spleens from various groups were collected, sliced into 2 mm × 2 mm segments, and treated with a digestive solution (consisting of 3% collagenase IV and 0.5% DNase I) at 37 °C for 2 h. The resultant cell suspensions were then labeled with specific antibody combinations as follows. Tumor tissues: MDSCs (anti-Gr-1-APC, anti-CD11b-PE), TAMs (anti-F4/80-APC, anti-CD206-PE), CD4^+^ and CD8^+^ T cells (anti-CD3-PE, anti-CD4-FITC, anti-CD8-APC). TDLN: mature DCs (anti-CD11c-APC, anti-CD80-FITC, anti-CD86-PE), exhausted T cells (Ex T) (anti-CD3-FITC, anti-CD8-APC, anti-TCF-1-PE). Spleen: Tregs (anti-CD4-FITC, anti-CD25-APC, anti-FoxP3-PE), CD4^+^ and CD8^+^ T cells (anti-CD3-PE, anti-CD4-FITC, anti-CD8-APC), central memory T cells (T_CM_) and effector memory T cells (T_EM_) (anti-CD8-PE, anti-CD44-FITC, anti-CD62L-APC).

### Immunohistochemistry assay

On the 17th day, the mice were euthanized, and both tumor tissues and major organs were collected. Subsequently, all the tumor tissues and organs were encased in paraffin and sliced into sections of 4 μm in thickness. These sections were then subjected to an incubation process involving primary antibodies, secondary antibodies, and DAPI staining, after which they were examined using an inverted microscope Eclipse Ts2 (Nikon, Tokyo, Japan). For histological examination, hematoxylin and eosin (HE) staining was conducted on sections of both the tumor and major organs.

### Cytokines

The release of antitumoral cytokines such as interferon-γ (IFN-γ), interleukin-10 (IL-10), and interleukin-12 (IL-12) within the tumors following each treatment was monitored through the utilization of ELISA kits. After 3 treatments, tumor tissues from each treatment group were meticulously gathered and homogenized for the subsequent measurements.

### Serum biochemical analysis

At the end of the experiment, the mice were euthanized under anesthesia, and blood samples were obtained by means of intracardiac puncture. Subsequent to centrifugation (4000 rpm, 15 min), the plasma was extracted and preserved at − 80 °C. The levels of aspartate aminotransferase (AST), blood urea nitrogen (BUN), alanine aminotransferase (ALT), and creatinine (CRE) were assessed utilizing a Cobas 6000 automatic biochemical analyzer (Roche, Switzerland).

### Lung metastasis model

1 × 10^7^ 4T1 cells were subcutaneously implanted into the right mammary fat pad. When the tumor volume exceeded 90 mm^3^, mice bearing 4T1 tumors were randomly allocated into four treatment cohorts (1: Control; 2: Free CUR; 3: CUR@DD-Hb NPs; 4: CUR/miR155@DD-Hb NPs, n = 3). Subsequently, 2 × 10^6^ 4T1 cells were intravenously injected via the tail vein to obtain lung metastasis model. Treatment regimens were administered every two days for a total of five doses. Following the completion of treatments, the lungs of the mice were harvested, fixed using Bouin's solution, and evaluated for the presence, size, and distribution of metastatic tumors.

### Statistical analysis

At least three paralleled experiments were conducted, and the results are presented as the mean ± standard deviation (SD). Statistical comparisons were performed by the oneway analysis of variance (ANOVA) among the three groups. Paired comparisons were made by Student’s t-test between two groups. A p value < 0.05 was considered to have statistical significance.

## Results and discussion

### Synthesis and characterization of copolymer

The Gu, Hb and DSMA monomers were synthesized according to the literature [29, 30]. To synthesize the macroinitiator mPEG-CTA, CPADB, which possessed a carboxyl terminus, was connected to mPEG-OH through an esterification reaction. Subsequent reversible addition–fragmentation chain transfer (RAFT) copolymerization of Gu, DSMA, and Hb monomers resulted in the formation of PEG-P(Gu)-P(DSMA)-P(Hb) with mPEG-CTA acting as the macroinitiator. Characterization through ^1^H NMR spectroscopy was employed to confirm the chemical structures of the compounds, verifying the successful synthesis of these materials (Figure S2–6).

The hemolysis assessment demonstrated minimal hemoglobin release subsequent to exposure to PEG-P(Gu)-P(DSMA)-P(Hb) at a concentration of 2 mg/mL, contrasting with the outcomes of exposure to PBS. These results suggest auspicious biocompatibility and safety for intravenous delivery (Figure S7).

### Characterization of nanocomplexes

**Scheme 1:**
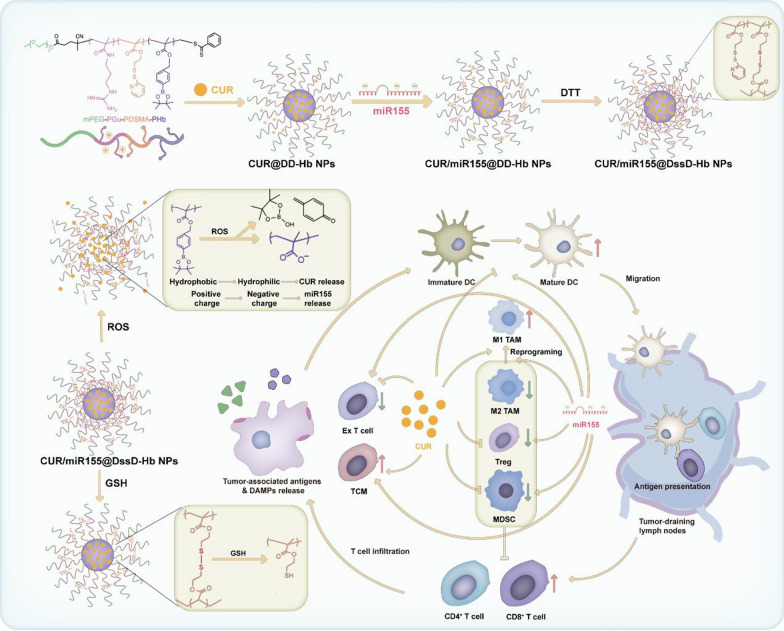
Illustration of ROS/GSH responsive nanoparticles loaded with CUR and miR155 for targeting tumor and reshaping the tumor microenvironment

**Fig. 1:**
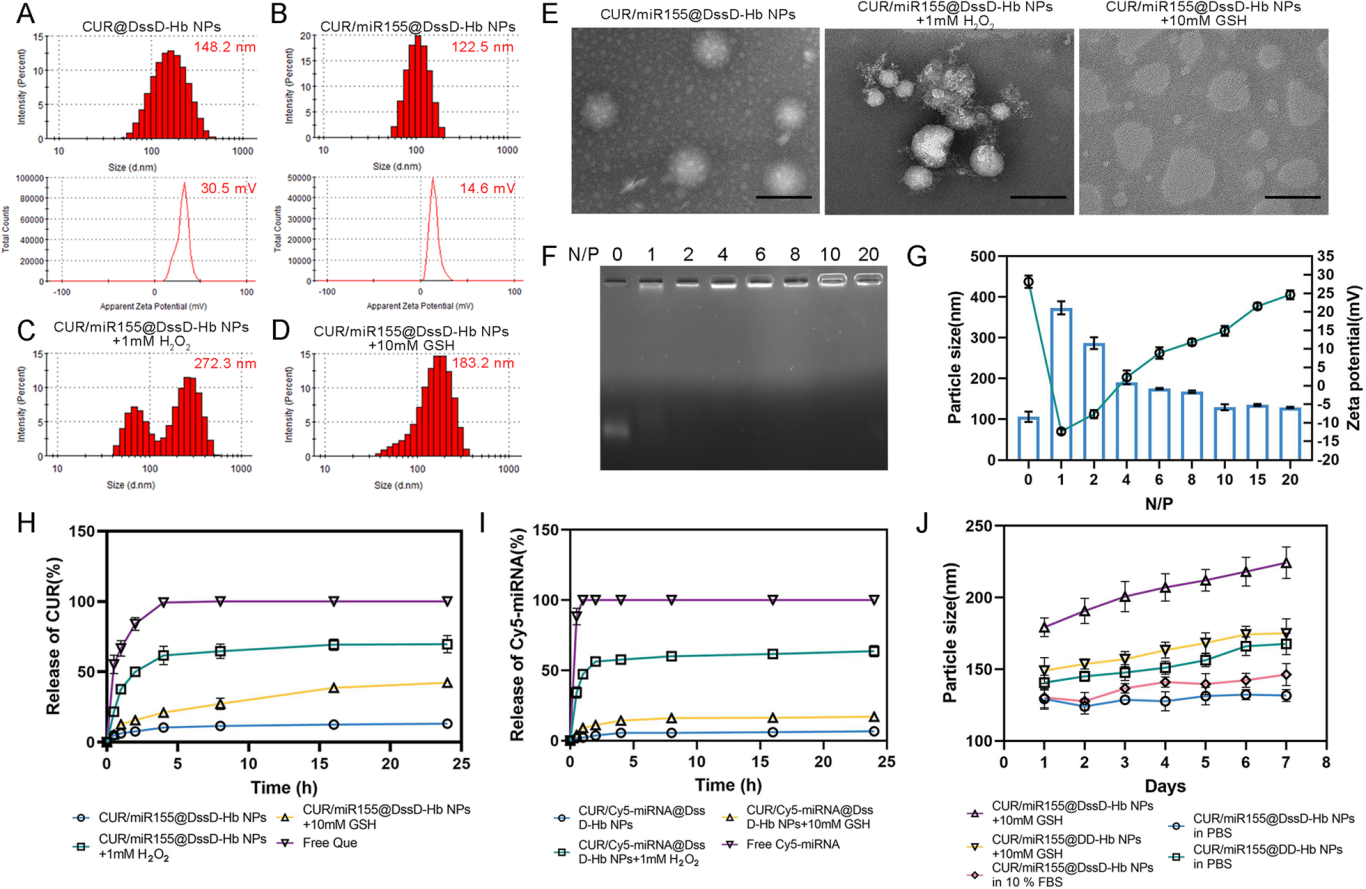
The physiochemical properties of CUR/miR155@DssD-Hb NPs. Representative particle size and zeta potential results of CUR@DssD-Hb NPs (**A**) and CUR/miR155@DssD-Hb NPs (**B**). Particle size of CUR/miR155@DssD-Hb NPs after 1 mM H_2_O_2_ (**C**) and 10 mM GSH (**D**) treatment. **E** The TEM results of CUR/miR155@DssD-Hb NPs, CUR/miR155@DssD-Hb NPs treated with 1 mM H_2_O_2_ and CUR/miR155@DssD-Hb NPs treated with 10 mM GSH. Scale bar = 200 nm. **F** miRNA binding ability of CUR/miR155@DssD-Hb NPs by the agarose gel retardation assay. **G** Particle sizes and zeta potential of CUR/miR155@DssD-Hb NPs at different N/P molar ratios. Release profiles of CUR (**H**) and Cy5-miRNA (**I**) from CUR/miR155@DssD-Hb NPs in different release media under 37 ℃. **J** The particle size change of different formulations in different media

The process of self-assembly was shown in Scheme 1. mPEG-P(Gu)-P(DSMA)-P(Hb) as amphiphilic copolymer can self-assemble into shell-core nanoparticles through hydrophobic interaction. The CUR is entrapped into the hydrophobic core of the nanoparticles while miR155 can be loaded via electrostatic interactions. Considering the substantial influence of physicochemical properties on the behavior of nanocomplexes in vitro and in vivo, we conducted a comprehensive investigation of various parameters including particle size, morphology, zeta potential, EE, LE, stability, and drug release behavior in the presence of the ROS and GSH. The DLS results revealed that the hydrodynamic diameter of CUR@DssD-Hb NPs was 148.2 nm (Fig. 1A). Through optimization of the Gu and miR155 N/P ratio to 10:1 (Fig. 1F and G), the preferred CUR/miR155@DssD-Hb NPs were selected for further investigation with their optimal sizes of 122.5 nm (Fig. 1B), moderate surface charges of 14.6 mV, homogeneous and compact morphologies captured via TEM imaging (Fig. 1E), and effective miR155 encapsulation. As shown in Table 1, the LE % values of CUR and miR155 were determined to be 6.75% and 3.63%, respectively, indicative of the efficient loading of CUR and miR155.

**Table 1:** Characterization of Blank@DssD-Hb NPs, CUR@DssD-Hb NPs and CUR/miR155@DssD-Hb NPs (n = 3)

Formulations	Size (nm)	Zeta potential (mV)	EE (%)	LE (%)
CUR@DssD-Hb NPs	145.23 ± 4.21	30.87 ± 0.44	72.43 ± 5.62% (CUR)	6.75 ± 0.43% (CUR)
CUR/miR155@DssD-Hb NPs (N/P = x)	121.56 ± 2.28	15.35 ± 1.28	98.36 ± 2.15% (miR155)	3.63 ± 0.25% (miR155)

For the successful delivery of siRNA to tumor sites post systemic administration, nano-delivery systems must maintain structural integrity evade nucleases, and mitigate protein adsorption. Upon incubation of CUR/miR155@DssD-Hb NPs with 10% FBS, a minor increase in particle size of less than 20 nm was observed, suggesting robust stability. An ideal drug carrier should remain stable within a simulated normal physiological environment but be capable of disassociating in a simulated tumor tissue environment. The disulfide cross-linked structure provides enhanced stability, preventing premature drug leakage in physiological conditions. When CUR/miR155@DssD-Hb NPs encounter the elevated levels of ROS typically found in cancer cells, the hydrophobic Hb component undergoes conversion to its hydrophilic form bearing carboxyl groups. This transition from hydrophobic to hydrophilic state initially weakens the hydrophobic stabilization and subsequently the newly introduced carboxyl groups interfere with electrostatic interactions. This gradual “self-destruct” mechanism facilitates efficient release of CUR and mi155. The uncrosslinked CUR/miR155@DD-Hb NPs showed the large size of 145.8 nm, while crosslinked CUR/miR155@DssD-Hb NPs displayed little smaller particle diameter, with a size of 122.5 nm. The stability experiments demonstrated that the size of the CUR/miR155@DssD-Hb NPs remained relatively constant in PBS within 7 d. In contrast, the size of the CUR/miR155@DD-Hb NPs exhibited a significant increase of ~ 20 nm during the same time frame. Once CUR/miR155@DssD-Hb NPs were treated with GSH (10 mM), the particle size of CUR/miR155@DD-Hb NPs significantly increased. These findings highlighted the superior stability conferred by the cross-linked disulfide linkage.

Given the sensitivity of phenylboronic esters to ROS, we investigated the particle size of CUR/miR155@DD-Hb NPs under the influence of the ROS-inducing agent H_2_O_2_. Illustrated in Fig. 1C, the incubation of 1 mM H_2_O_2_ prompted the breakdown of CUR/miR155@DssD-Hb NPs, resulting in a substantial rise in particle dimensions (~ 272.2 nm at 6 h). This alteration in particle size was further corroborated by TEM images (Fig. 1E). CUR/miR155@DssD-Hb NPs maintained their spherical shapes and pristine morphology in the absence of H_2_O_2_. Contrastingly, when H_2_O_2_ was introduced, these nanoparticles exhibited swelling, eventually adopting a hollow spherical structure. This phenomenon could potentially be attributed to the oxidation of the borate components within the hydrophobic Hb to hydrophilic carboxyl groups upon exposure to H_2_O_2_.

The in vitro drug release behavior was investigated via dialysis (for CUR) and the ultrafiltration method (for Cy5-miRNA) (Fig. 1H and I). Leveraging the presence of a disulfide bond, we posited that cross-linked structure could impede the premature seepage of CUR and miR155 in a physiological environment. As depicted in Fig. 1H and I, free CUR and Cy5-miRNA displayed rapid release into medium with 95% detectable within 4 h. Conversely, upon encapsulation within CUR/Cy5-miRNA@DssD-Hb NPs, a notable reduction in the release of CUR and Cy5-miRNA was observed, with only 10% and 5% observed after 48 h, indicative of robust stability and devoid of any abrupt discharges. Prompted by the dissociation of disulfide bonds, the 48 h-cumulative release percentages of CUR and siRNA from CUR/Cy5-miRNA@DssD-Hb NPs in the presence of 10 mM GSH were higher than those in the absence of GSH. The ROS-sensitive drugs release was investigated and the results indicated H_2_O_2_ treatment enabled rapid CUR and Cy5-miRNA release from CUR/Cy5-miRNA@DssD-Hb NPs in a controllable way, likely attributable to the disassembly of nanoparticles arising from the hydrophobic-to-hydrophilic shift of Hb.

### Cellular uptake and cytotoxicity of CUR/miR155@DssD-Hb NPs in vitro.

**Fig. 2:**
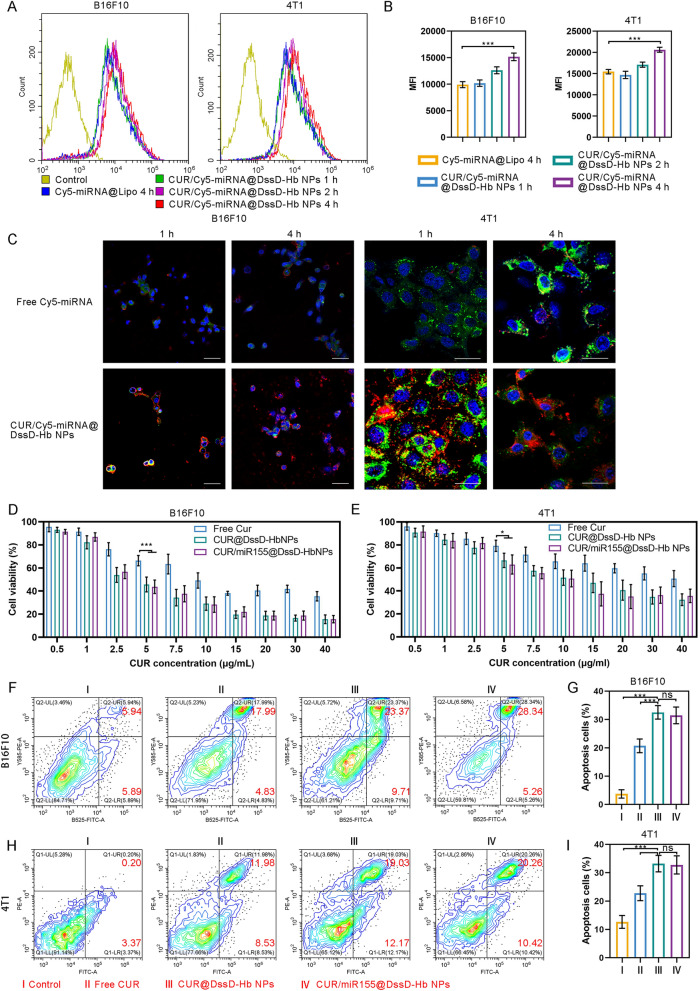
In vitro cellular uptake and cytotoxicity. Flow cytometry (**A**) and quantification (**B**) of cellular uptake in 4T1 and B16F10 cells treated with different CUR preparations at 1, 2 and 4 h. **C** CLSM observation of 4T1 and B16F10 cells incubated with Cy5-miRNA-loaded nanoparticles at 1 and 4 h. Red: Cy5-miRNA; blue: nucleus; green: mitochondria. Scale bar = 50 μm. The comparison of cytotoxicity in B16F10 (**D**) and 4T1 (**E**) cells. Apoptosis and analysis of B16F10 (**F** and **G**) and 4T1 (**H** and **I**) cells after various formulations of treatment for 24 h. (n = 3. ns, no significance, *p < 0.05, **p < 0.01 and ***p < 0.001)

Typically, the therapeutic efficacy of many nanomedicines hinge on their internalization and subsequent intracellular release within cancer cells [31]. This holds particularly true for nucleotides like miRNA, where the negative charge elicits repulsion against the cell membrane, impeding its passive diffusion. Overcoming intracellular barriers, such as endosomal and lysosomal degradation, is imperative for ensuring the efficient therapeutic impact of miRNA [31]. CLSM was employed to elucidate cellular uptake and intracellular trafficking (Fig. 2C). The results showed that the internalization of free Cy5-miRNA by 4T1 and B16F10 cells was barely detectable at 1 and 4 h. On the contrast, upon the initial uptake of CUR/miR155@DssD-Hb NPs by 4T1 and B16F10 cells within 1 h, obvious yellow dots can be detected, arising from colocalization of Lyso-Tracker (green) and Cy5-miRNA (red). After 4 h, these yellow signals dissipated, suggesting the successful release of Cy5-miRNA from the lysosomes. This phenomenon led to a reasonable postulation that the nanocomplexes facilitated escape from endosomes/lysosomes and the cytosolic release of miRNA through the "proton sponge" effect of Gu [29]. Flow cytometry analysis (Fig. 2A and B) further confirmed the enhanced cellular uptake of CUR/miR155@DssD-Hb NPs in B16F10 and 4T1 cells facilitated by the ROS- and GSH-sensitive groups, exhibiting a 1.53- and 1.33-fold increase in cellular uptake compared to Cy5-miRNA@Lipo at 4 h. In summary, CUR/Cy5-miRNA@DssD-Hb NPs bolstered the cellular uptake of miRNA and fostered the intracellular release of miRNA.

To further investigate the cytotoxic potential of various formulations, in vitro cell cytotoxicity assays were conducted on B16F10 and 4T1 cells utilizing the MTT assay. As shown in Fig. 2D, CUR/miR155@DssD-Hb NPs and CUR@DssD-Hb NPs markedly repressed B16F10 and 4T1 tumor cell proliferation in a dosage-dependent fashion. Notably, CUR/miR155@DssD-Hb NPs exhibited stronger suppressive activity against B16F10 cells compared to 4T1 cells. The inhibitory activity of CUR/miR155@DssD-Hb NPs revealed minimal variance from CUR@DssD-Hb NPs in both B16F10 and 4T1 cells. To evaluate the cytotoxicity of CUR/miR155@DssD-Hb NPs on normal cells, HEK293 cells were employed in this investigation (Figure S8A). Furthermore, Blank@DssD-Hb NPs exhibited negligible cytotoxic effects on B16F10 and 4T1 cells at 100 μg/mL (Figure S8B and C). These findings underscored the safety of the nanoparticles at the experimental concentration as carriers for CUR and miR155 delivery.

### Cell apoptosis

Moreover, the apoptotic population of cells treated with different formulations was assessed through flow cytometry utilizing Annexin V-FITC and PI staining. Illustrated in Fig. 2G, the proportion of 4T1 cells treated with CUR@DssD-Hb NPs (5 μg/mL) in the apoptotic state was 31.20%, much higher than that of free CUR (20.51%) and control group (3.57%). Notably, the apoptosis-inducing capacity of CUR/miR155@DssD-Hb NPs showed negligible difference (30.68%) compared with CUR@DssD-Hb NPs, aligning with the observations from the MTT assay. At the same concentration, CUR/miR155@DssD-Hb NPs elicited a 33.60% overall apoptosis rate in B16F10 cells, marking a 1.51-fold increase compared to the free CUR group. These outcomes underscored that the efficacious delivery of CUR could drive apoptosis in both B16F10 and 4T1 cells.

### ROS production

Numerous studies have illustrated that curcumin induces heightened levels of intracellular ROS, leading to oxidative damage and apoptosis in tumor cells [32]. Hence, the impact of CUR formulations on ROS production in 4T1 and B16F10 tumor cells was investigated by FCM, employing DCFH-DA as a fluorescent indicator. As depicted in Figure S9, flow cytometry results revealed that both free CUR and CUR-loaded formulations (CUR@DssD-Hb NPs and CUR/miR155@DssD-Hb NPs) augmented ROS levels at a concentration of 5 μg/mL of CUR in 4T1 and B16F10 cells. Furthermore, CUR@DssD-Hb NPs magnified the ability of curcumin to elevate ROS levels, achieving a 7.3 and 4.7-fold enhancement in 4T1 and B16F10 cells, possibly attributable to the enhanced cellular uptake of CUR. CUR/miR155@DssD-Hb NPs showcased similar ROS-inducing capabilities. These findings indicated that CUR/miR155@DssD-Hb NPs could stimulate ROS generation mediated by CUR as a ROS initiator in tumor cells, potentially hastening nanoparticle disintegration and CUR release, thereby establishing a positive feedback loop to bolster antitumor efficacy.

### In vitro migration and invasion assay

Research has elucidated that miR-155 can either promote or hinder the migration and invasion of various cancer cell lines through the posttranscriptional suppression of mRNA [33, 34]. The impact of CUR/miR155@DssD-Hb NPs on migration and invasion was examined using wound-healing and transwell invasion assays. As illustrated in Figure S10, free CUR and CUR@DssD-Hb NPs treatment (CUR: 2 μg/mL) exhibited the capacity to impede the migration of 4T1 and B16F10 cells, which might be attributed to down-regulation of JAK-2/STAT3 signaling pathway. Notably, CUR/miR155@DssD-Hb NPs manifested the most potent anti-migratory effects among the test groups in 4T1 cells, while demonstrating comparable wound healing rates to CUR@DssD-Hb NPs in B16F10 cells. Research has indicated that the upregulation of miR-155 can curb the invasive and migratory potential of 4T1 tumors by suppressing the expression of the transcription factor TCF4, a pivotal regulator of EMT [34]. In the transwell assay (Figure S10D), a marked decrease in the number of invaded B16F10 and 4T1 cells was observed in both the CUR@DssD-Hb NPs and CUR/miR155@DssD-Hb NPs groups when compared with the control group. Consequently, the administration of CUR could potentially serve as a promising strategy in combating melanoma and TNBC metastasis.

### ICD

**Fig. 3:**
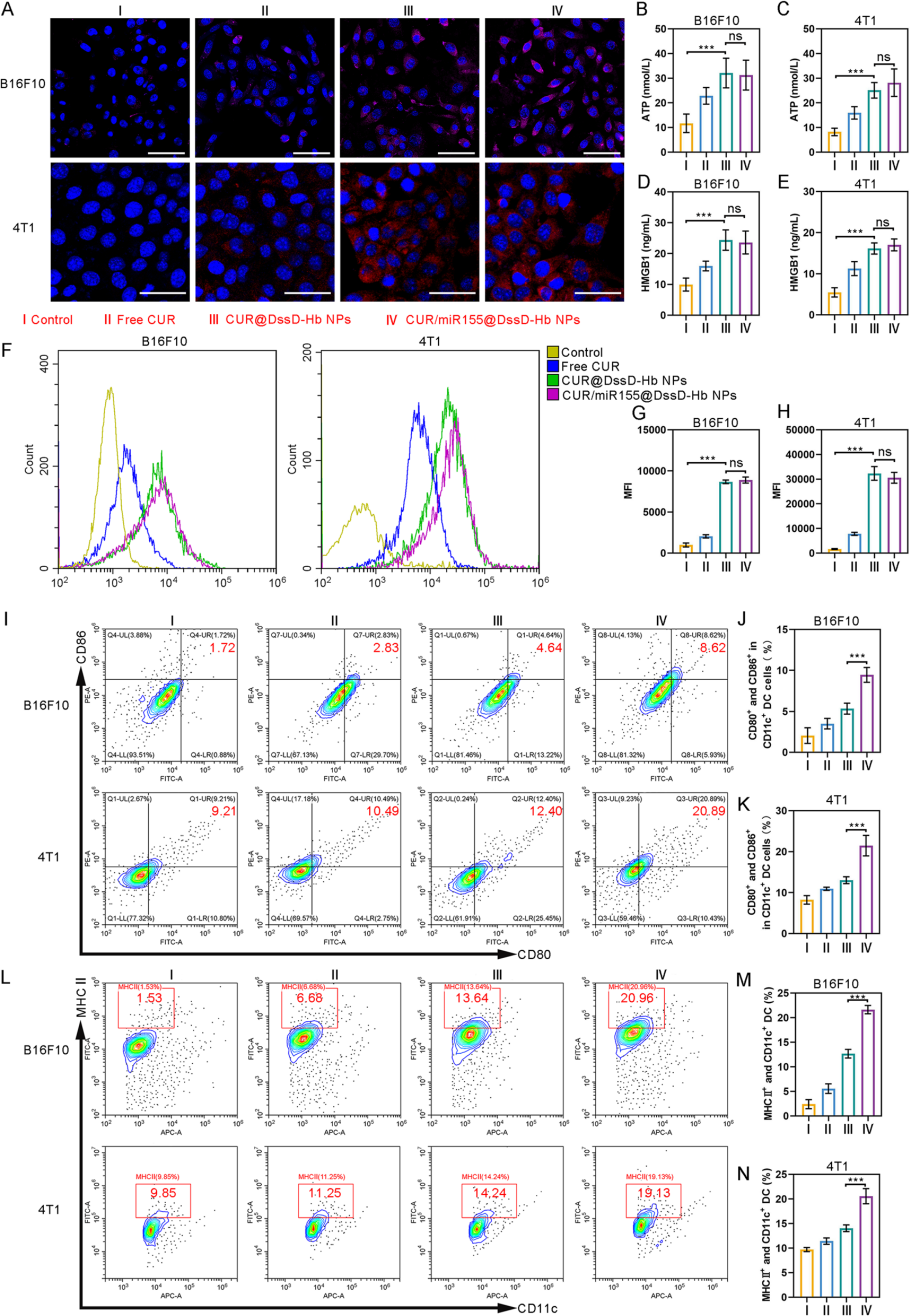
The induction of ICD in 4T1 and B16F10 cells and the maturation of BMDCs. **A** CLSM of CRT eversion in 4T1 and B16F10 cells after different treatments. B16F10: Scale bar = 50 μm. 4T1: Scale bar = 25 μm. Extracellular ATP (**B** and **C**) and HMGB1 (**D** and **E**) level of 4T1 and B16F10 cells after different treatments. The flow cytometry (**F**) and corresponding quantitative analysis (**G** and **H**) of CRT expressions after treatment. **I**–**K** The expression of CD80 and CD86 in BMDCs was analyzed by flow cytometry (gated on CD11c^+^ cells). **L**–**N** The expression of MHC II in BMDCs was analyzed by flow cytometry. (n = 3. ns, no significance, *p < 0.05, **p < 0.01 and ***p < 0.001)

ICD is widely recognized as a crucial component for effective cancer immunotherapy. Several studies have indicated that CUR can induce ICD through autophagy [35–38]. The process of ICD involves the release of damage associated molecular patterns (DAMPs), including CRT, ATP, and HMGB1. These signals can stimulate the maturation of DCs, subsequently facilitating the activation of T cells. The externalization of CRT was observed through CLSM and flow cytometry. CLSM showed that the treatment of CUR@DssD-Hb NPs and CUR/miR155@DssD-Hb NPs dramatically promoted the exposure of CRT with stronger fluorescence intensity than free CUR and control in B16F10 and 4T1 cells (Fig. 3A) [35, 37]. The flow cytometry results (Fig. 3F, G and H) were consistent with the CLSM results. The levels of HMGB1 and ATP in the supernatant were quantified via ELISA. The findings demonstrated that CUR@DssD-Hb NPs and CUR/miR155@DssD-Hb NPs substantially augmented the secretion of HMGB1 and ATP. Conversely, treatment with free CUR resulted in a bit of HMGB1 and ATP release from B16F10 and 4T1 cells (Fig. 3B–E). Moreover, there were no significant distinctions observed between the CUR@DssD-Hb NPs and CUR/miR155@DssD-Hb NPs groups regarding CRT externalization and the release of HMGB1 and ATP in both B16F10 and 4T1 cells.

### Maturation and cross-presentation analysis of BMDCs

DCs, as a major antigen-presenting cells (APC), play a pivotal role in initiating, regulating, as well as maintaining innate and adaptive immune responses. The induction of ICD by CUR/miR155@DssD-Hb NPs may stimulate the maturation of DCs in vitro via the release of "eat me" signals and tumor-associated antigens. Nonetheless, within tumor-bearing hosts, DCs often exhibit an immature and malfunctioning phenotype due to the limited tumor immunogenicity and the presence of immunosuppressive agents secreted by either tumor cells or tumor-associated immunosuppressive cells, thereby allowing tumors to evade immune surveillance [39, 40]. Moreover, curcumin has been known to prompt a tolerogenic state in DCs by hindering the expression of maturation markers, cytokines, and chemokines, thereby impeding the response of DCs to immune-stimulating agents. [41]. On the contrary, several studies have indicated that miR155 can transition DCs from a tolerogenic state to an immune-activated condition, triggering robust anti-tumor responses, thereby suggesting that upregulating miR-155 expression could notably enhance the effectiveness of DC-based immunotherapies [42]. Therefore, we hypothesized that miR155 might attenuate the suppressive impacts on DCs induced by CUR, ultimately leading to a more efficacious anti-tumor immune response. As shown in Fig. 3I–K, BDMCs were incubated with drug-pretreated 4T1 and B16F10 cells, and the expression of surface costimulatory molecules CD80 and CD86 were analyzed by flow cytometry to evaluated the maturation of DCs. Free CUR-treated 4T1 cells and B16F10 exhibited a modest ability to stimulate DCs maturation. B16F10 treated with CUR@DssD-Hb NPs exhibited a heightened capacity to trigger DCs maturation. Although CUR had an inhibitory effect on DCs maturation, the release of tumor-associated antigens and DAMPs induced by CUR stimulated DCs maturation to a certain extent [37, 43]. miR155 further augmented this process, as evidenced by the notable immunogenicity induced by B16F10 cells treated with CUR/miR155@DssD-Hb NPs, resulting in nearly 8.62% DCs maturation, surpassing the 4.64% seen in the CUR@DssD-Hb NPs group (Fig. 3I). The combination of miR155 and CUR (CUR/miR155@DssD-Hb) also achieved the highest promotion of DCs maturation in 4T1 cells. At the same time, treatment with CUR/miR155@DssD-Hb NPs led to significantly increased proportions of CD11c^+^ and MHC-II^+^ BMDCs in both 4T1 and B16F10 cells compared to other treatment groups, signifying an enhancement in the competence of antigen cross-presentation (Fig. 3L–N).

### In vivo biodistribution

**Fig. 4:**
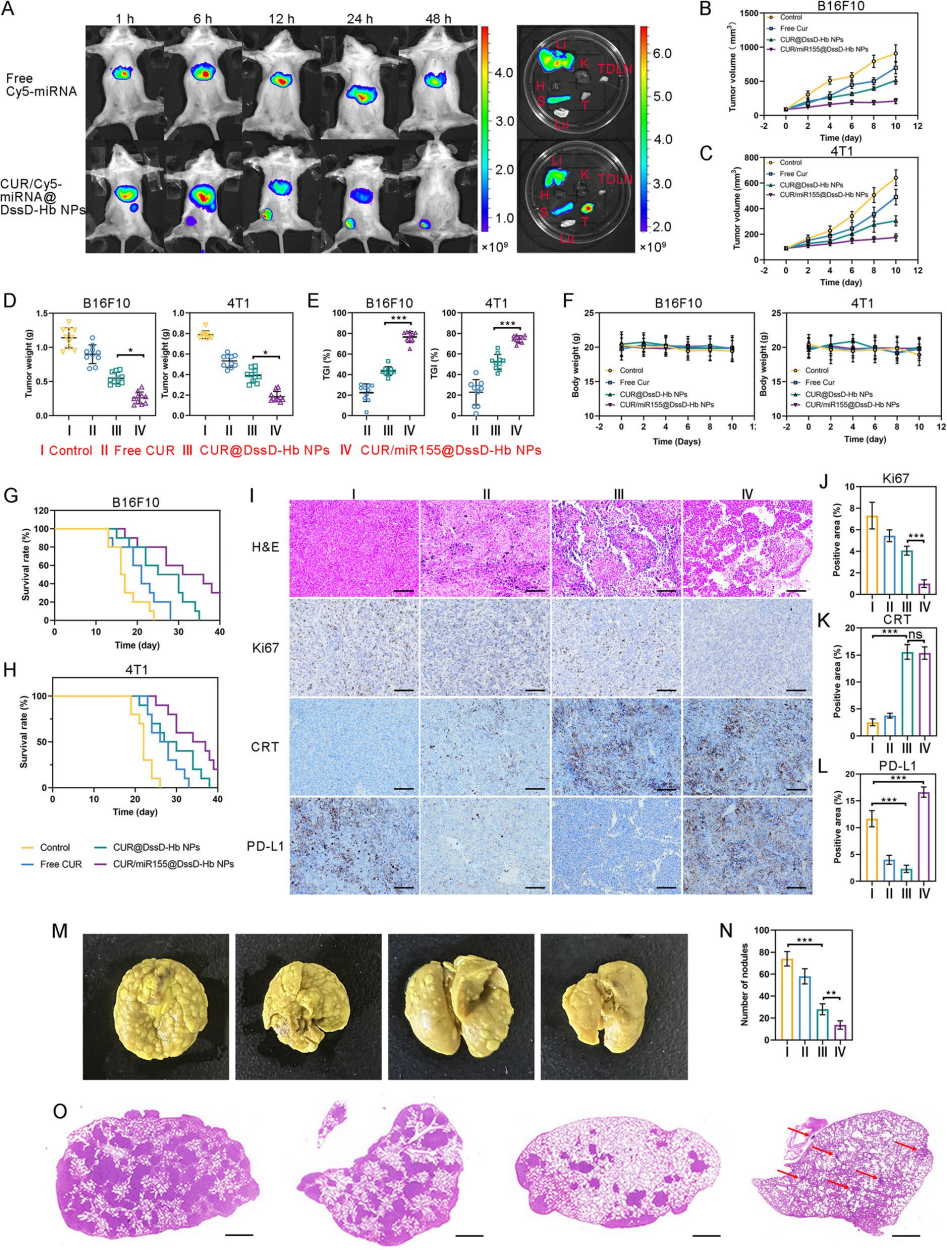
In vivo biodistribution, antitumor and anti-metastasis evaluation. **A** In vivo biodistribution of Cy5-labeled nanocomplexes in 4T1 inoculation mice after intravenous administration. Fluorescence images of excised major organs and tumor tissue after mice sacrifice at 48 h after intravenous administration. T: tumor; H: heart; Lu: lung; Li: liver; S: spleen; K: kidney; TDLN: tumor draining lymph node. Tumor growth curves (**B**, **C**) of B16F10 and 4T1 tumor-bearing mice in different groups during administration. The tumor weight (**D**), TGI (**E**) of B16F10 and 4T1 tumor-bearing mice in different groups. **F** Body weight of B16F10 and 4T1 tumor-bearing mice in different groups during administration. Kaplan–Meier survival curves of B16F10 (**G**) and 4T1 (**H**) tumor-bearing mice received different treatments (n = 10). **I**–**L** H&E, Ki67, CRT and PD-L1 staining of B16F10 tumor slides. **M** Representative images of lung metastasis. **N** Quantification analysis of lung metastatic nodules. **O** H&E staining of lung slides. Lung metastatic nodules are denoted by red arrows. (n = 3, *p < 0.05, **p < 0.01 and ***p < 0.001)

Nanoparticles ranging between 50 and 200 nm in size possess the ability to passively accumulate within tumor tissues through enhanced permeability and retention effect (EPR) effect, facilitated by damage vessel structure and lymphatic system [44]. In addition, ROS and GSH sensitive groups can enable CUR/miR155@DssD-Hb NPs to release cargos in tumor site via responding to tumoral stimuli. CUR/Cy5-miRNA@DssD-Hb NPs instead of CUR/miR155@DssD-Hb NPs were used to investigated the biodistribution of NPs in vivo. As shown in Fig. 4A, during the initial hours, fluorescence signals from free Cy5-miRNA and CUR/Cy5-miRNA@DssD-Hb NPs were detected in the liver. At 6 h, the fluorescent signal emanating from CUR/Cy5-miRNA@DssD-Hb NPs began to appear at the tumor sites, with the intensity of the signal progressively increasing with time. The ex vivo fluorescence images further confirmed that DiR@DssD-Hb group had much higher Cy5-miRNA accumulation in tumor site compared with free Cy5-miRNA group. There results indicated the effective delivery of drugs into tumors by CUR/miR155@DssD-Hb NPs.

### In vivo antitumor effect of CUR/miR155@DssD-Hb NPs

Given the excellent performance of CUR/miR155@DssD-Hb NPs in vitro, Balb/c and C57BL/6 mice were employed to establish 4T1 and B16F10 tumor-bearing mouse models to assess the therapeutic potential of CUR/miR155@DssD-Hb NPs. Free CUR exhibited only moderate inhibition of tumor growth compared to the control group. In B16F10 tumor-bearing mice, the relative tumor volume in the CUR@DssD-Hb NPs group was significantly lower than that in the free CUR group, indicating that the tumor growth inhibitory effect of CUR could be enhanced by the nanoplatform (Fig. 4B). Furthermore, the co-delivery of CUR and miR155 into the tumor led to a more potent suppression of tumor growth, with the tumor weight measuring 0.287 g in the CUR/miR155@DssD-Hb NPs group (Free CUR: 0.895 g, CUR@DssD-Hb NPs: 0.493 g), suggesting that miR155 played a critical role in tumor eradication. The tumor growth curve in 4T1-tumor bearing mice following various treatments, shown in Fig. 4C, exhibited a similar trend to that in B16F10 tumor-bearing mice.

To further investigate the histological alterations and the expression of tumor-related proteins in tumor tissues, H&E staining and immunofluorescence staining were conducted on the B16F10 tumor tissues. The H&E staining revealed significant levels of necrotic and apoptotic cell death with dense nuclear pyknosis and cytoplasmic karyorrhexis in tumors treated with CUR/miR155@DssD-Hb NPs and CUR@DssD-Hb NPs, aligning with their outstanding antitumor efficacy. In the Ki67 assay, the least amount of Ki67-positive cells (appearing brown-yellow) was observed in the CUR/miR155@DssD-Hb NPs group, signifying that CUR/miR155@DssD-Hb NPs could effectively inhibit the proliferation of tumor cells.

The remarkable anti-tumor efficacy of CUR/miR155@DssD-Hb NPs may be attributed to the direct cell-killing effect of CUR and the reprogramming of the immunosuppressive TME facilitated by miR155 and CUR. The initiation of ICD triggered by CUR in B16F10 solid tumors was validated by the exposure of CRT. As shown in Fig. 4I, negligible CRT remained to be observed with control and free CUR groups in B16F10 and 4T1 tumor sections. On the contrary, CUR/miR155@DssD-Hb NPs and CUR@DssD-Hb NPs increasingly facilitated CRT expression of B16F10 tumor cells, suggesting substantial tumor cell death with immunogenic properties. These DAMPs enhance the presence of mature DCs, which present DAMPs and tumor-specific antigens to T cells, activating CD8^+^ CTLs.

PD-L1 is expressed on tumor cells and controls lifespan of cytotoxic CD8^+^ T cells when interacting with PD-1 [45]. Recent research highlights how heightened PD-L1 levels lead to diminished T-cell functionality and consequent evasion of the immune system by tumors [46]. As shown in Fig. 4I, CUR@DssD-Hb NPs significantly reduced the expression of PD-L1 in B16F10 tumor tissue. CUR effectively suppressed the IFN-γ-triggered elevation of PD-L1 by impeding STAT1 phosphorylation, thereby furnishing a mechanistic elucidation for its modulation of PD-L1 in melanoma cells [47]. On the contrary, a significant positive correlation was found between the miR-155 concentration and the extent of PD-L1 expression in melanoma, breast cancer, glioma and B cell lymphomas tumor tissue [13, 18, 48–50]. CUR/miR155@DssD-Hb NPs induced higher expression of PD-L1 compared to CUR@DssD-Hb NPs, implying its potential in harnessing the efficacy of immune checkpoint blockade (ICB) therapy [18].

Moreover, no significant fluctuations in serum biochemical indicators or histological changes in major organs were observed in B16F10 tumor-bearing mice across all treatment groups compared to the control group, indicating the safety of the drug delivery system (Figure S11). The combination of tumor progression suppression and minimal side effects with CUR/miR155@DssD-Hb NPs contributed to an improved survival rate in tumor-bearing mice (Fig. 4G and H). These findings strongly implied that our synergistic formulation bears promising antitumor capabilities with minimal side effects, stemming from the combination of CUR and miR155, paving the way for future clinical antitumor therapies.

### Lung metastases

Due to the potent in vitro anti-metastasis ability of CUR/miR155@DssD-Hb NPs, we have the reason to believe that CUR/miR155@Bio/PE-NPs as a potential drug delivery strategy can prevent TNBC metastasis in vivo. A mouse model for breast cancer lung metastasis was established to evaluate the anti-metastasis activity in vivo. The expression of miR-155 displayed an inverse correlation with assorted EMT markers in TNBC tissues [34, 51]. In addition, CUR exerts an anti-metastasis effect through the suppression of JAK-2/STAT3 signaling pathway. As shown in Fig. 4M–O, a substantial accumulation of metastatic nodules within the lungs was observed in the control group, whereas CUR administration (CUR@Bio/PE-NPs) demonstrated an obvious reduction. This effect was further enhanced by miR155 (CUR/miR155@Bio/PE-NPs), exhibiting the lowest number of nodules.

### In vivo remodeling of TME

**Fig. 5:**
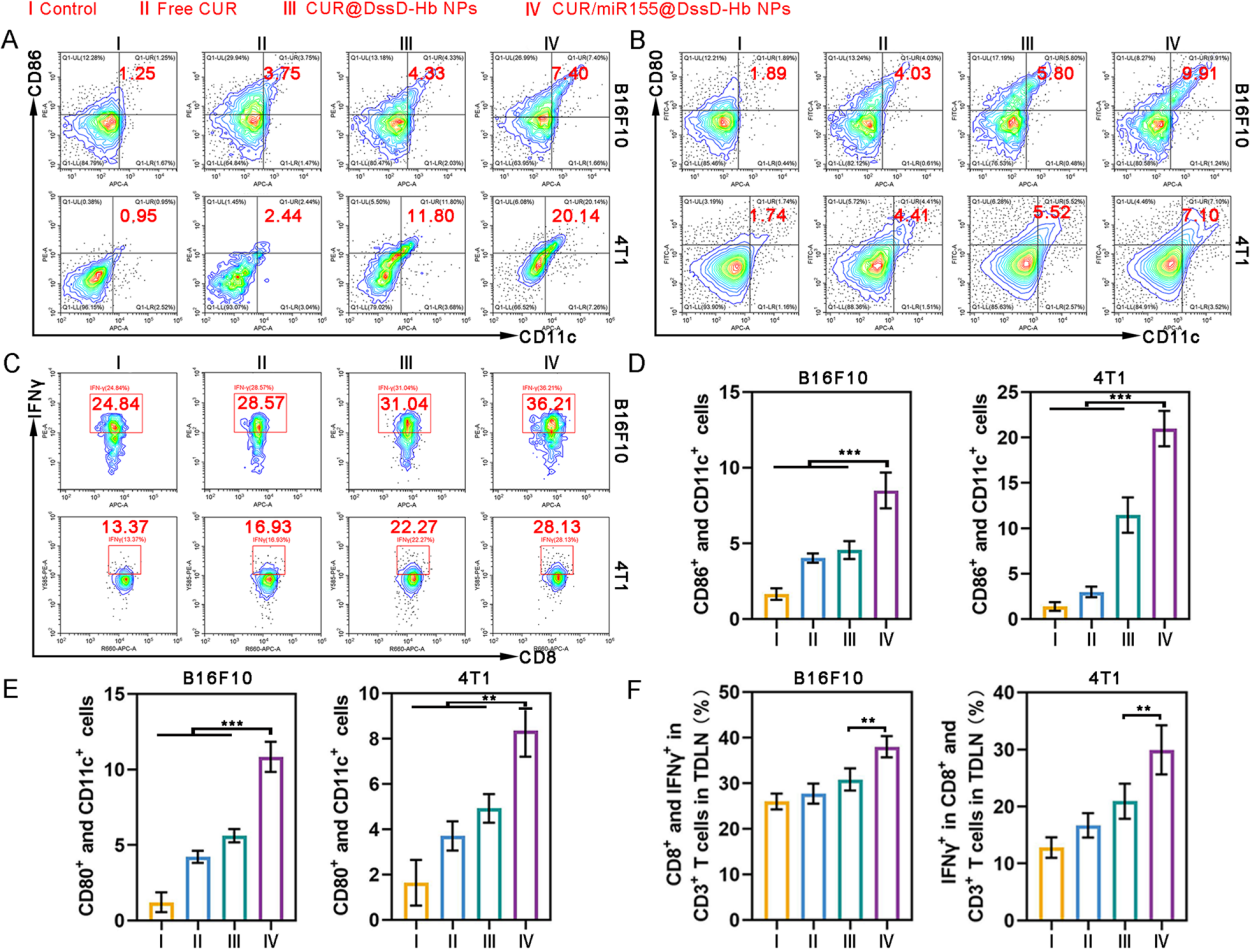
Immune responses within TDLNs. **A** and **B** Representative flow cytometric analysis of the proportion of mature DCs in TDLN. **C** Representative flow cytometric analysis of IFN-γ^+^ CD8^+^ T cells in TDLNs. The quantification analysis of CD11c^+^ CD86^+^ (**D**), CD11c^+^ CD80^+^ (**E**) and IFN-γ^+^ CD8^+^ cells (**F**) in TDLNs. (n = 3, **p < 0.01 and ***p < 0.001)

To elucidate the intricate antitumor immune mechanisms underlying the exceptional antitumor efficacy of CUR/miR155@DssD-Hb NPs, it is imperative to observe the migration of mature DCs from the tumor site to the TDLNs, where they can activate cytotoxic T lymphocytes against cancer. The levels of co-stimulatory molecule expression (CD80^+^ and CD86^+^) in CD11c^+^ DCs isolated from the TDLNs were measured. As depicted in Fig. 5A and B, the CD86^+^ and CD80^+^ DCs (among CD11c^+^ cells) within the CUR/miR155@DssD-Hb NPs group displayed the most obvious upregulation compared to all other groups in both B16F10 and 4T1 tumor-bearing murine models, aligning with the in vitro results of BMDCs stimulation. Notably, the introduction of miR155 in CUR/miR155@DssD-Hb NPs augmented the ability to induce DCs maturation when compared with the CUR@DssD-Hb NPs group. These mature DCs potentially stem from intratumoral DCs incited by miR155, thereby affording abundant opportunities for cross-presentation and cross-priming of CD8^+^ T cells. To examine the priming status of CD8^+^ T cells, the number of IFN-γ-producing CD8^+^ T cells within the TDLNs was quantified via flow cytometry. As depicted in Fig. 5C and F, CUR/miR155@DssD-Hb NPs elicited a heightened presence of IFN-γ^+^ CD8^+^ T cells, demonstrating a 1.16- and 1.26-fold increase over that of CUR@DssD-Hb NPs in B16F10 and 4T1 tumor-bearing mice, respectively [18]. Our findings suggest that DCs activated within the tumor by CUR/miR155@DssD-Hb NPs might migrate to the TDLNs, thereby bolstering immune responses.

**Fig. 6:**
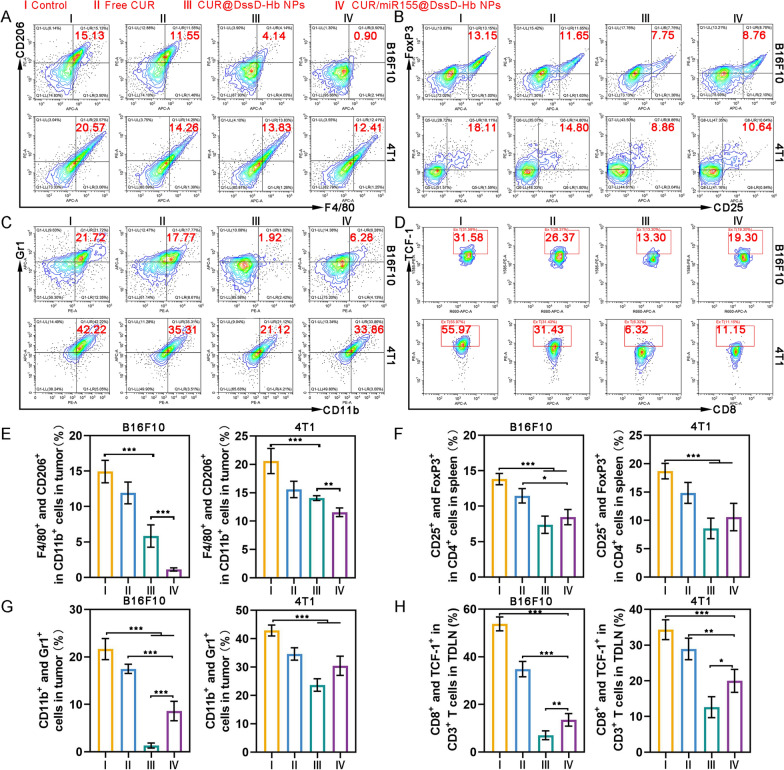
Immunosuppressive cell components analysis. Representative flow cytometric analysis of the proportion of M2 TAMs (F4/80^+^ and CD206^+^ in CD11b^+^ cells) (**A**) in tumor, Tregs (CD25^+^ and FoxP3^+^ in CD4^+^ cells) (**B**) in spleen, MDSCs (CD11b^+^ and Ly6G^+^) (**C**) in tumor, and Ex T (CD8^+^ and TCF-1^+^ in CD3^+^ cells) (**D**) in TDLN. The quantification analysis of M2 TAMs (**E**), Tregs (**F**), MDSCs (**G**) and Ex T (**H**). (n = 3, *p < 0.05, **p < 0.01 and ***p < 0.001)

The expansion of immunosuppressive cellular populations, including Tregs, MDSCs, and TAMs, serves as a pivotal stratagem employed by tumors to elude detection and elimination by the immune system. Consequently, the modulation of the immunosuppressive milieu is emerging as a fundamental approach to substantially transform “cold” tumors into “hot” tumors, thereby amplifying the effectiveness of cancer immunotherapy. Within the TME, M2 TAMs stand out as the principal immunosuppressive cellular entities, contributing to the production of pro-tumor factors, recruitment of Tregs, and dysfunction of tumor-killing effector cells such as CD8^+^ T and NK cells. Elevated expression of miR-155 in TAMs triggers the reprogramming of pro-tumoral M2 TAMs into an M1 anti-tumoral phenotype [10]. Furthermore, curcumin has the capacity to modulate various tumor mediators and cytokines, leading to the reprogram of pro-tumoral M2 TAMs into M1 [52]. The administration of CUR@DssD-Hb NPs led to a reduction in the M2 TAMs population, showing a decrease of approximately 10.99% and 6.74% in B16F10 and 4T1 tumor models, respectively, compared to those in the control group (Fig. 6A). Notably, CUR/miR155@DssD-Hb NPs exhibited enhanced inhibitory efficacy on M2 TAMs (Fig. 6H). M2 TAMs have the capacity to amplify the proliferation of other immunosuppressive cell types such as Tregs and MDSCs [52, 53]. While miR155 has been linked to the accumulation of Tregs and MDSCs in tumors [22], curcumin has been shown to target Tregs, converting them into Th1, and to impede the recruitment and build-up of MDSCs within the TME [25]. In the B16F10 tumor model, the percentage of Tregs within tumors following treatment with CUR@DssD-Hb NPs and CUR/miR155@DssD-Hb NPs was found to be 7.75% and 8.76%, respectively, marking a decrease when compared to the 13.15% seen in the control group (Fig. 6B and F). Additionally, CUR/miR155@DssD-Hb NPs exhibited a reduction in the recruitment of MDSCs compared to both the control group and the free CUR group (Fig. 6C and G). Likewise, in 4T1 tumor-bearing mice, CUR/miR155@DssD-Hb effectively depleted Tregs and MDSCs within the tumors. These outcomes underscored their potential in reshaping the immunosuppressive TME.

In cancer patients, long-term exposure to persistent antigens and inflammation leads to continual stimulation of T cells. Over time, these T cells undergo exhaustion, losing their effector function and gradually losing their memory T cell characteristics. Liu et al. demonstrated that curcumin alleviated T cell exhaustion by inhibiting the PD-1 and TIM-3 axes in both CD4^+^ and CD8^+^ T cells [54]. On the contrary, increased expression of miR-155 facilitated the expansion and prolonged presence of Ex T cells [55]. The CUR/miR155@DssD-Hb NPs group exhibited the lower ratio of Ex T cells in both 4T1 and B16F10 tumor-bearing mice, compared to free CUR and control group (Fig. 6D and H). This underscores that the CUR/miR155@DssD-Hb NPs proves advantageous for the restoration of T cell functionality [56, 57].

**Fig. 7:**
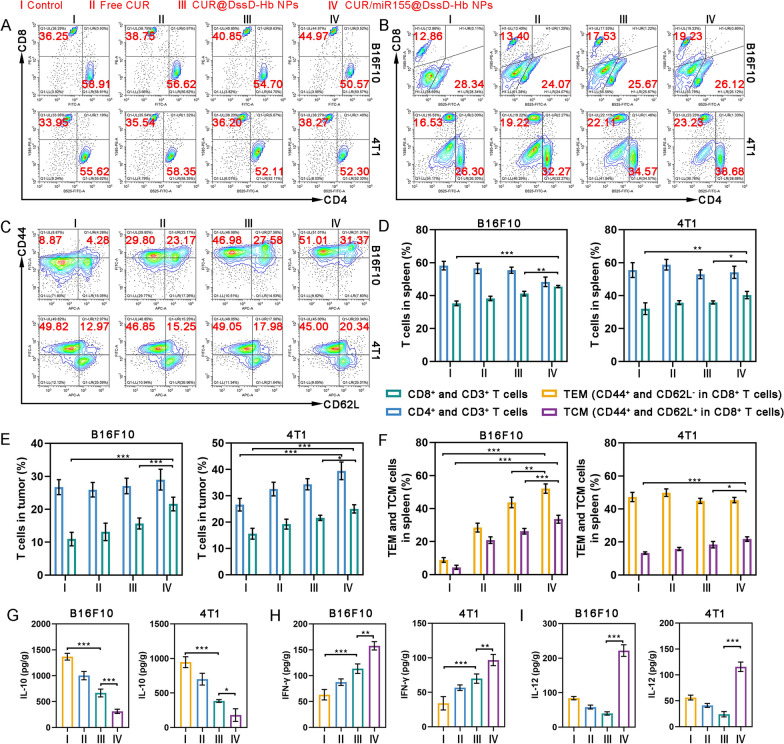
In vivo immune response after different treatments. Representative flow cytometric analysis and the proportion of CD4^+^ and CD8^+^ in CD3^+^ T cells in spleen (**A**) and tumor (**B**). **C** Representative flow cytometric analysis and the proportion of T_EM_ (CD44^+^ and CD62L^−^ in CD8^+^ T cells) and T_CM_ (CD44^+^ and CD62L^+^ in CD8^+^ T cells) in spleen. The quantification analysis of CD4^+^ and CD8^+^ in CD3^+^ T cells in spleen (**D**) and tumor (**E**) and T_EM_ and T_CM_ in spleen (**F**). The concentrations of IL-10 (**C**), IFN-γ (**D**) and IL-12 (**I**) in the B16F10 and 4T1 tumor. (n = 3, *p < 0.05, **p < 0.01 and ***p < 0.001)

The assessment of T lymphocyte cells was conducted to ascertain the potential of CUR/miR155@DssD-Hb NPs in activating an effective cellular immune response. The percentage of CD3^+^ and CD8^+^ T cells in spleen treated with CUR/miR155@DssD-Hb NPs had elicited a highest peak frequency (Fig. 7A and D). The ratio of CD8^+^ T cells was close to 44.97% and 38.27% in B16F10 and 4T1 tumor bearing mice, almost 1.24 and 1.13 times than control, indicating the positive impact of the synergistic therapy based on miR155 and CUR in bolstering systemic immune responses.

The recruitment and infiltration of T cells into tumors play a pivotal role in the antitumor immune response, indicating their immediate tumor-killing potential. Meanwhile, overexpression of miR-155 is known to enhance the proliferation and effector function of CD8^+^ T cells [20]. In both B16F10 and 4T1 tumor-bearing mice, a notable 1.36-fold and 1.34-fold increase, respectively, in the distribution of CD8^+^ T cells was observed in the CUR@DssD-Hb NPs group compared to the control (Fig. 7B and E). Furthermore, CUR/miR155@DssD-Hb NPs facilitated a heightened recruitment and infiltration of CD8^+^ T cells into tumors, reaching 19.23% and 23.23% in B16F10 and 4T1 tumors, respectively. These outcomes strongly support the establishment of a successful immune cascade, laying the groundwork for enhanced immunotherapy.

Moreover, the maintenance of antitumor immune memory is vital for long-term prevention of tumor recurrence. Memory T cells are broadly categorized into T_CM_ and T_EM_. By analyzing spleens from various treatment groups (Fig. 7C and F), it was found that the CUR/miR155@DssD-Hb NPs group in B16F10 tumor mice exhibited a notable increase in the proportion of T_CM_ compared to the control and CUR@DssD-Hb NPs group, with a similar trend observed in 4T1 tumors, indicating a successful preservation of long-term immunological memory against tumors [12, 58, 59].

The secretion of IL-10, IL-12 and IFN-γ in tumor tissue were monitored. IL-10 has the capacity to hinder DCs maturation by suppressing miR-155 upregulation [17]. Therefore, targeted removal of IL-10 through CUR treatment may unleash the full potential of DCs in triggering antitumor immunity [26, 58, 60]. As depicted in Fig. 7G, the CUR/miR155@DssD-Hb NPs group exhibited the lowest level of IL-10 in B16F10 and 4T1 tumor tissues. Additionally, noticeable upregulation of IL-12 and IFN-γ was observed post CUR/miR155@DssD-Hb NPs treatment compared to CUR@DssD-Hb NPs. These variations in essential immune components suggested that the combination of CUR and miR155 has the ability to shift the balance from pro-tumor forces towards tumor-specific immunity.

## Conclusion

In conclusion, we constructed a ROS/GSH dual sensitive drug delivery system (CUR/miR155@DssD-Hb NPs) to co-delivery CUR and miR155 for activating the robust and long-lasting anti-tumor immune response. The characteristic tumor ROS triggers the transformation from hydrophobic (borate) to hydrophilic group (carboxyl), which promotes the release of CUR and miR155. GSH-sensitive PDSMA facilitates the crosslinking of themselves via disulfide bonds to improve stability and avoid the undesirable leakage during circulation. In vitro and in vivo results indicated CUR/miR155@DssD-Hb NPs can effectively inhibit 4T1 and B16F10 tumor cell viability, induce DAMPs release, promote the maturation of DCs and subsequent CD8^+^ T cell activation and infiltration, and deplete immunosuppressive cells, and reduce pulmonary metastatic nodules. The addition of CUR can able to minimize the immunosuppressive TME induced by miR155, therefore resulting in more effective anti-tumor immune response. Collectively, the synergistic combination of CUR and miR155 presents a promising immunotherapeutic approach for addressing melanoma and TNBC, holding significant potential for advancing treatment strategies in these malignancies.

### Supplementary Information

Additional file 1. Supplementary data to this article can be found online at: https://pubs.acs.org/doi/.

## Data Availability

The datasets used and/or analysed during the current study are available from the corresponding author on reasonable request.
