# Supplementary material for: Reactive oxygen species/glutathione dual sensitive nanoparticles with encapsulation of miR155 and curcumin for synergized cancer immunotherapy
Source: J Nanobiotechnology. 2024 Jul 8;22:400. doi: 10.1186/s12951-024-02575-5 (PMC11229347; doi:10.1186/s12951-024-02575-5)
Supplement: Supplementary file 1 — Additional file 1. Supplementary data to this article can be found online at: https://pubs.acs.org/doi/. [file 12951_2024_2575_MOESM1_ESM.docx]

**Reactive oxygen species/glutathione dual sensitive nanoparticles with encapsulation of miR155 and curcumin for synergized cancer immunotherapy**

Kangkang Li^a^, Juan Wang^b^, Yi Xie^a^, Ziyao Lu^a^, Wen Sun^a^, Kaixuan Wang^c^, Jinxin Liang^a^, Xuehong Chen^a*^

a School of Basic Medicine, Qingdao University, Qingdao, China

b Pharmacy Department, Qingdao Hospital, University of Health and Rehabilitation Sciences (Qingdao Municipal Hospital), Qingdao, China

c Department of Neurosurgery, Affiliated Hospital of Qingdao University, Qingdao, China

* Corresponding author

Xuehong Chen: chenxuehong@qdu.edu.cn

**Supplementary Material**


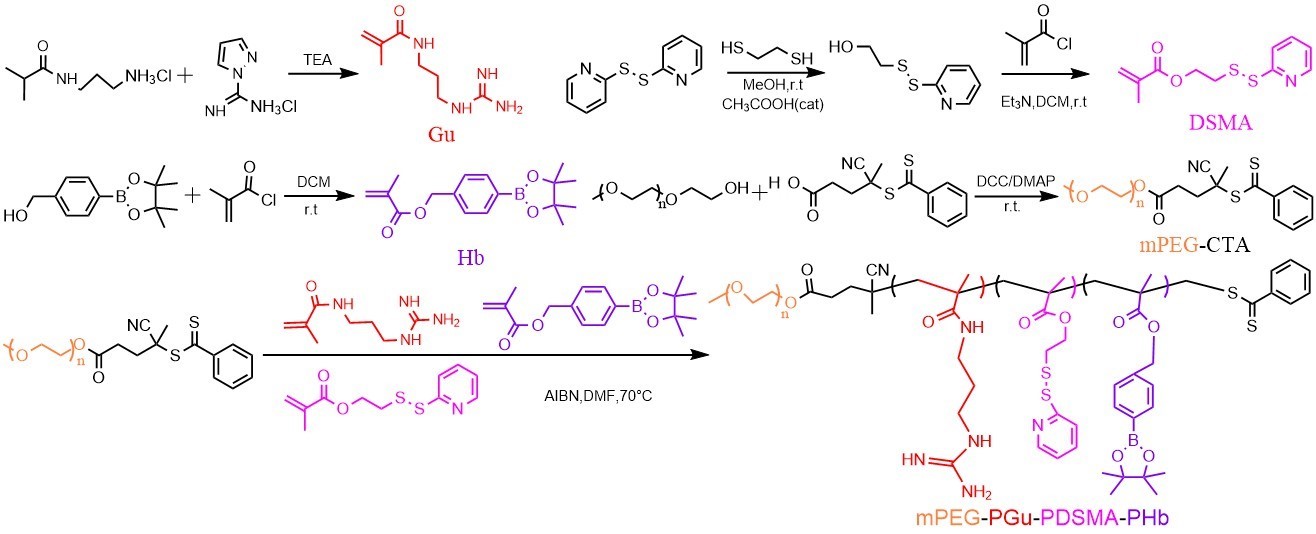


Figure S1. Synthesis route of mPEG-PGu-PDMSA-PHb.

**Synthesis of Gu**

APM (1 g, 5.60 mmol), praxadine (0.82 g, 5.60 mmol), Hydroquinone (10 mg) and TEA (1.24 g, 12.30 mmol) were dissolved in N,N-Dimethylformamide (DMF, 20 mL). and then stirred at r.t for 24 h under the protection of nitrogen atmosphere^1^. Subsequently, the mixture was poured into diethyl ether (80 mL). The precipitated oil was washed with acetonitrile (10 mL × 2), triethylamine (1 mL × 2) and DCM (20 mL × 2), respectively, and then dried by vacuo. The resultant pale-yellow sticky solid of Gu (0.44 g) was obtained with yield of 35.5%.

**Synthesis of DSMA**

Aldrithiol-2, 7 (15.0 g, 68.0 mmol) was dissolved in 75 mL of methanol and 1 mL of glacial acetic acid was added to it^2^. To this mixture, 15 mL methanol containing mercaptoethanol (2.7 g, 34.0 mmol) was added dropwise with continuous stirring. Subsequently, the reaction mixture was stirred at room temperature for 3 h. After removing the solvent by rotary evaporator, the crude product as yellow oil was purified by silica column chromatography (ethyl acetate/hexane). Compound was obtained as colorless oil (4.0 g, 61%).

After adding triethylamine (3 g, 29.7 mmol), the colorless oil was cooled to ~0 ℃. To this cold mixture, 10 mL DCM containing purified methacryloyl chloride (2.58 g, 24.7 mmol) was added dropwise with continuous stirring followed by stirring for 6 h at room temperature. After reaction finished, the mixture was washed with distilled water (30 mL × 3) and brine (30 mL × 2). The organic layer was collected, dried over anhydrous Na2SO4 and concentrated to get the crude product as yellow oil. It was purified by silica column chromatography (ethyl acetate/hexane). The DMSA ( mg) was obtained with yield of 80%.

**Synthesis of 4-(4,4,5,5-Tetramethyl-1,3,2-dioxaborolan-2-yl)benzyl methacrylate (Hb)**

4-(Hydroxymethyl)phenylboronic acid pinacol ester (2.5 g, 10.7 mmol) and TEA (1.3 g, 12.9 mmol) was dissolved in anhydrous DCM (20 mL) and cooled to ~ 0 ℃^1^. A solution of methacryloyl chloride (1.2 g, 11.0 mmol) in 2.5 mL dried DCM was dropwise added within 1 h. Once the addition was over, the reaction mixture was warmed to room temperature, stirred for 24 h and filtered. The filtrate was washed three times with brine. The production was dried with MgSO_4_ and purified by silica column chromatography (petroleum ether and ethyl acetate, v/v = 30/1). 4-(4,4,5,5-Tetramethyl-1,3,2-dioxaborolan-2-yl)benzyl methacrylate (Hb) was obtained as colorless liquid (2.5 g, yield:71.4 %).

**Synthesis of mPEG-CPADB**

mPEG2000 (5 g, 2.5 mmol), CPADB (2.79 g, 10 mmol) and DMAP (0.122 g, 1 mmol) were dissolved in 75 mL of anhydrous DCM and then stirred at 2 ℃ for 0.5 h. 25 mL anhydrous DCM containing DCC (2.06 g, 10 mmol) was added dropwise into the above mixture. The reaction was performed at 2 ℃ for 3 days followed by filtration. After removing the most of the DCM by rotary evaporator, the resultant mixture was precipitated in cold diethyl ether. The polymer was collected by filtration and the above precipitation procedure was repeated for three times. The residual solvent was removed in vacuo to give the crude product as a pink powder (4.6 g, 81.0%).

**Synthesis of mPEG-PGu-PDMSA-PHb**

The block copolymer mPEG-PGu-PDMSA-PHb was synthesized through RAFT. Briefly, mPEG-CPADB (0.1g, 0.05 mmol), Gu (0.05g, 0.26 mmol), Hb (0.1g, 0.33 mmol), DSMA (0.05g, 0.22mmol) and AIBN (0.005g, 0.03 mmol) were dissolved in 2 mL anhydrous DMF and sealed in a flask. After three freeze-pump-thaw cycles, the flask was sealed, and then polymerization was performed at 70 ℃ for 24 h under the protection of nitrogen. After the reaction finished, the production was precipitated in cold diethyl ether for three times and then dried by vacuo until constant weight (0.28 g, 93.3 %).

**Hemolysis test**

In order to assess the safety of nanocomplexes (e.g. through intravenous administration), hemocompatibility was examined. In summary, 2 mL of fresh rabbit blood containing heparin was diluted with PBS to a final concentration of 10 % prior to utilization. Next, 0.5 mL of CUR/miR155@NPs with varying concentrations were co-incubated with 0.5 mL of the aforementioned diluted blood for 2 h within a 37°C water bath, followed by centrifugation. Subsequently, the absorbance of the supernatant at 540 nm was gauged using a microplate reader (BIOTEK, ELX-800, USA).


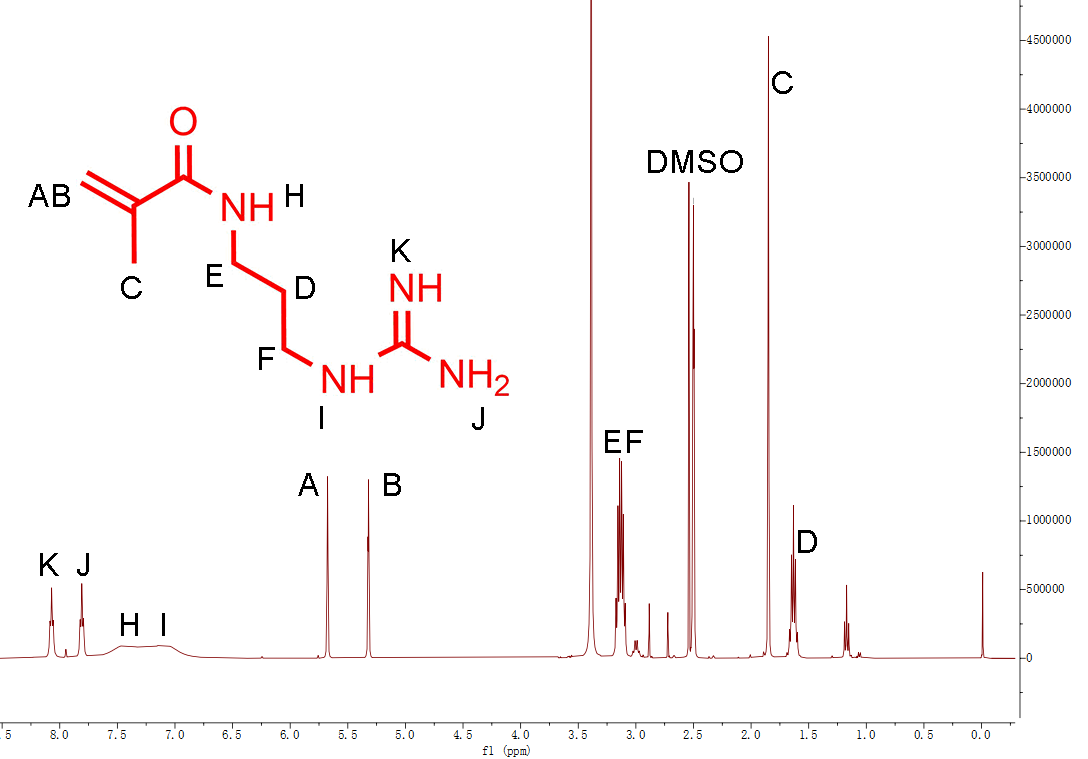


Figure S2. 1H NMR spectra of Gu.


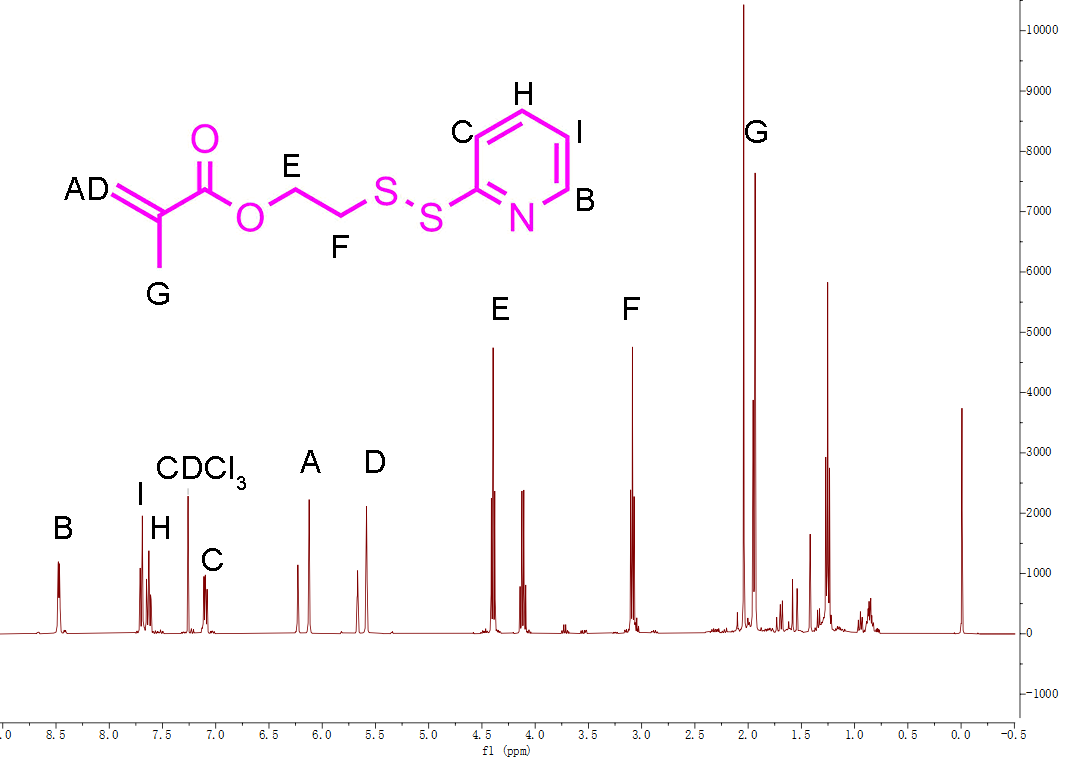


Figure S3. 1H NMR spectra of DSMA.


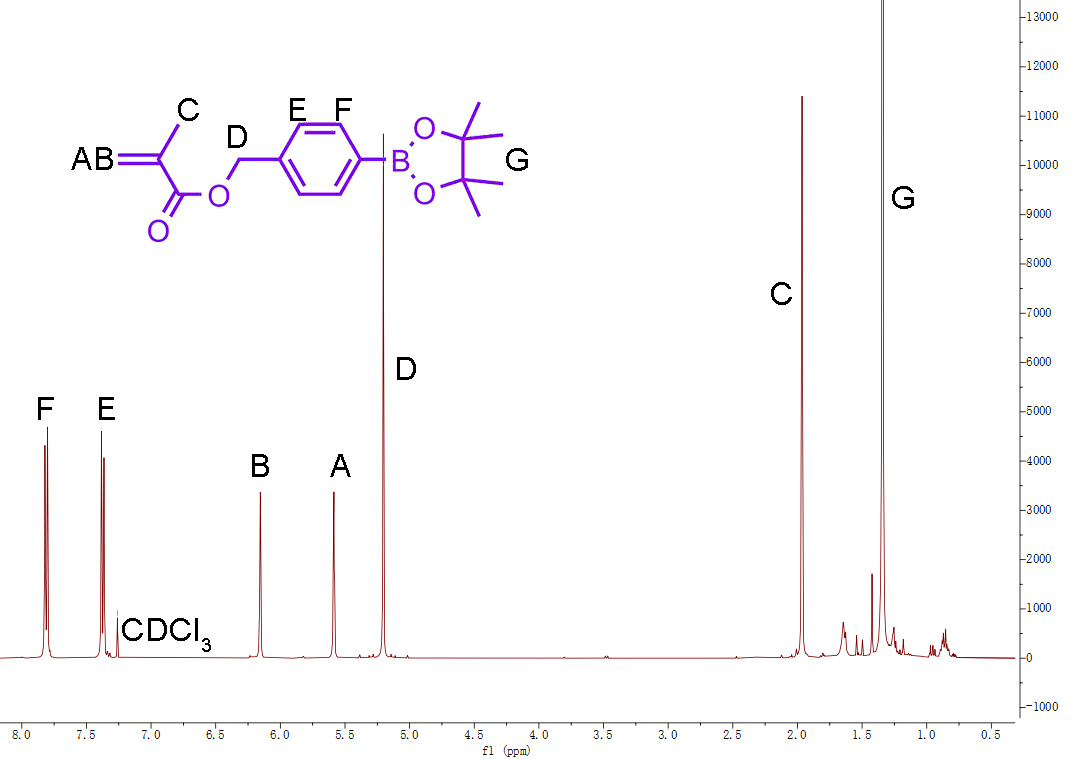


Figure S4. 1H NMR spectra of Hb.


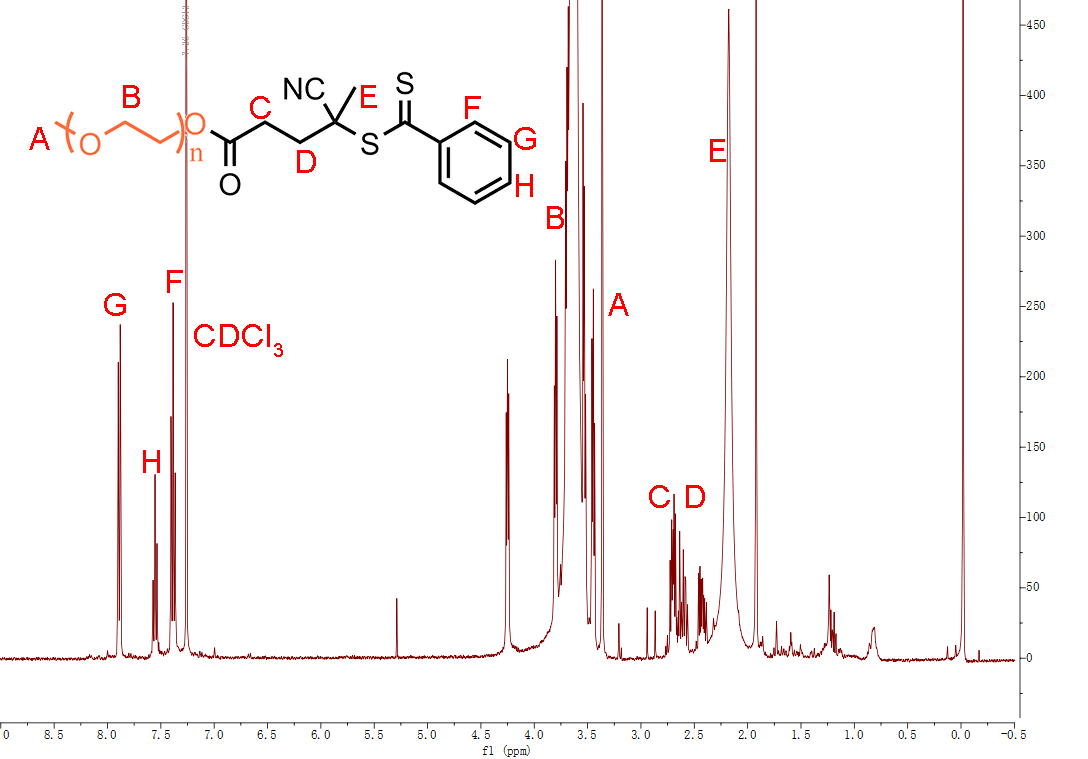


Figure S5. 1H NMR spectra of mPEG-CTA.


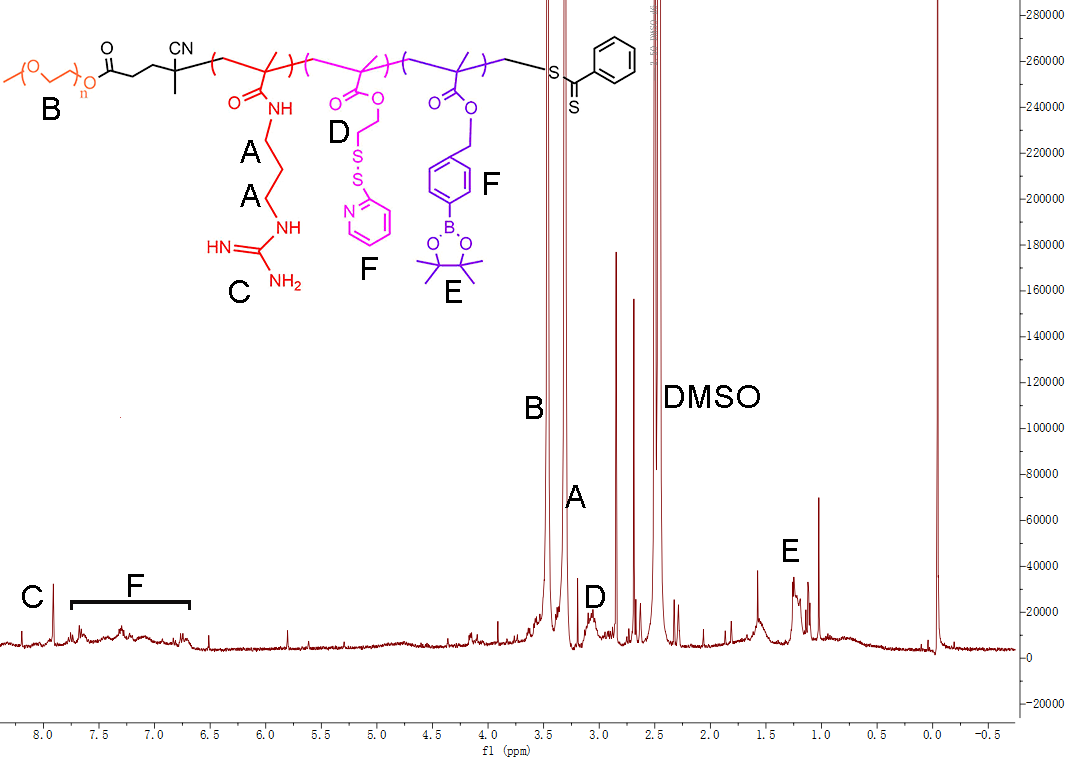


Figure S6. ^1^H NMR spectra of mPEG-PGu-PDMSA-PHb.


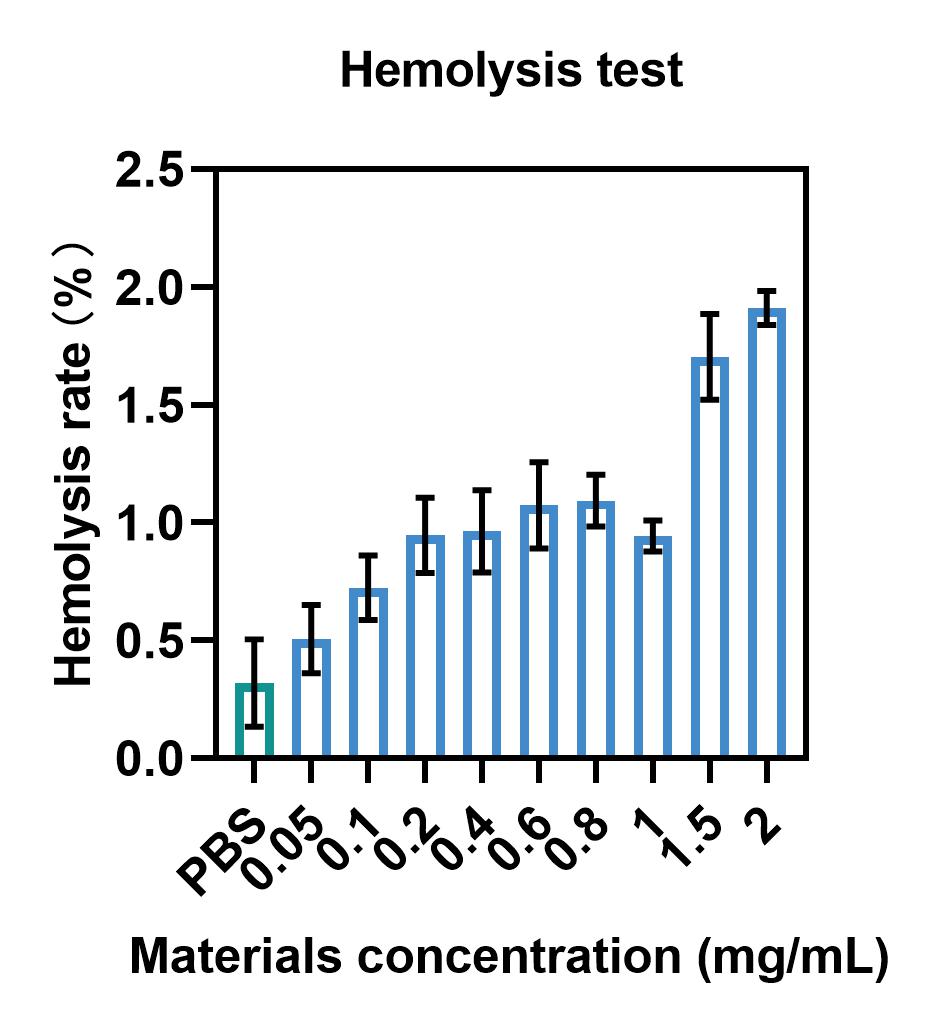


Figure S7. Hemolysis behaviors of red blood cells treated with PBS (negative control), and mPEG-PGu-PDMSA-PHb solution at concentration varying from 0.05 to 2.00 mg/mL (n = 3).


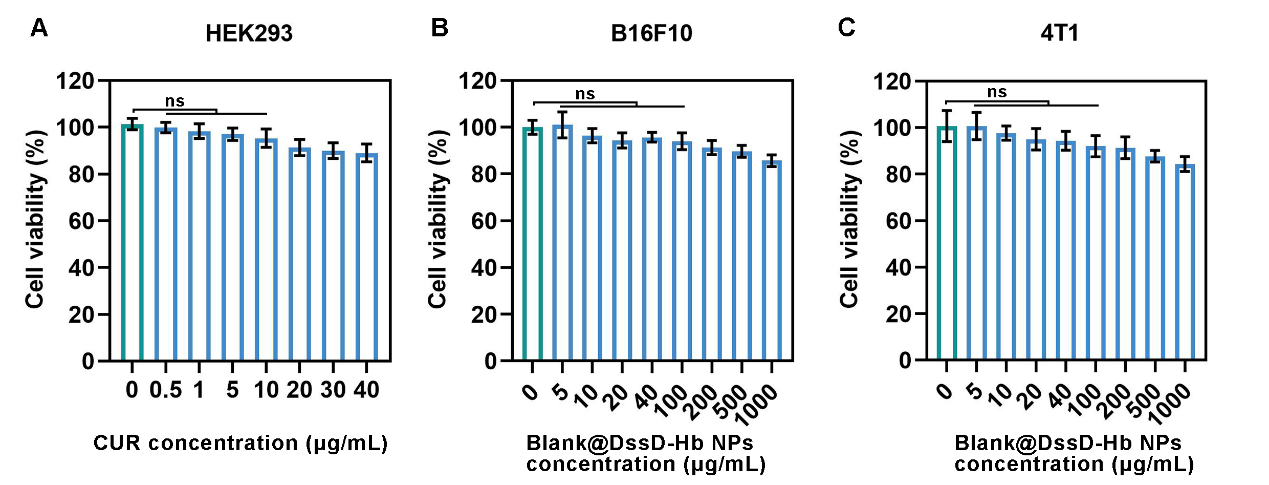


Figure S8. (A) The cytotoxicity of CUR/miR155@DssD-Hb NPs to HEK-293. The cytotoxicity of Blank@DssD-Hb NPs to B16F10 (B) and 4T1 (C) cells. (n = 3, ns, no significance.)


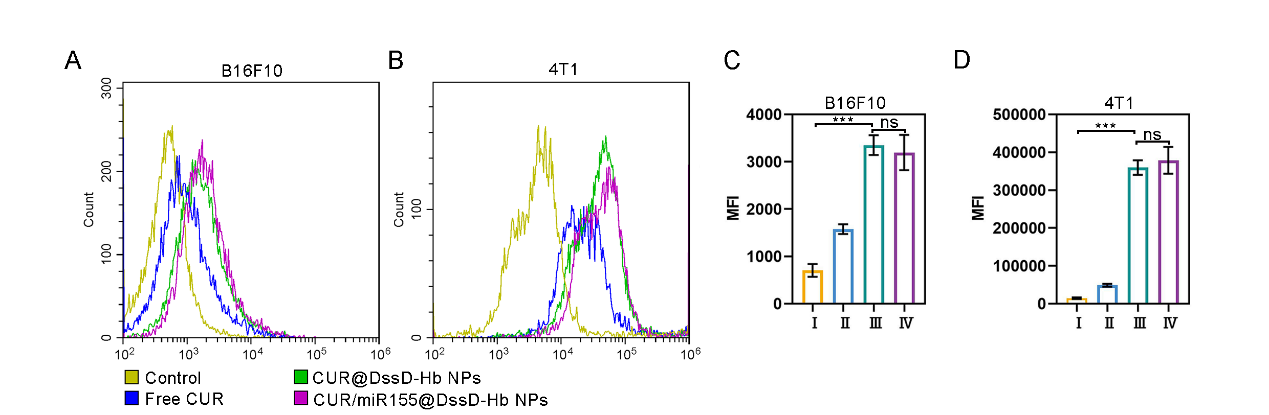


Figure S9. Flow cytometry and analysis of ROS production in 4T1 and B16F10 cells. (n = 3, ns, no, no significance, ***P < 0.001.)


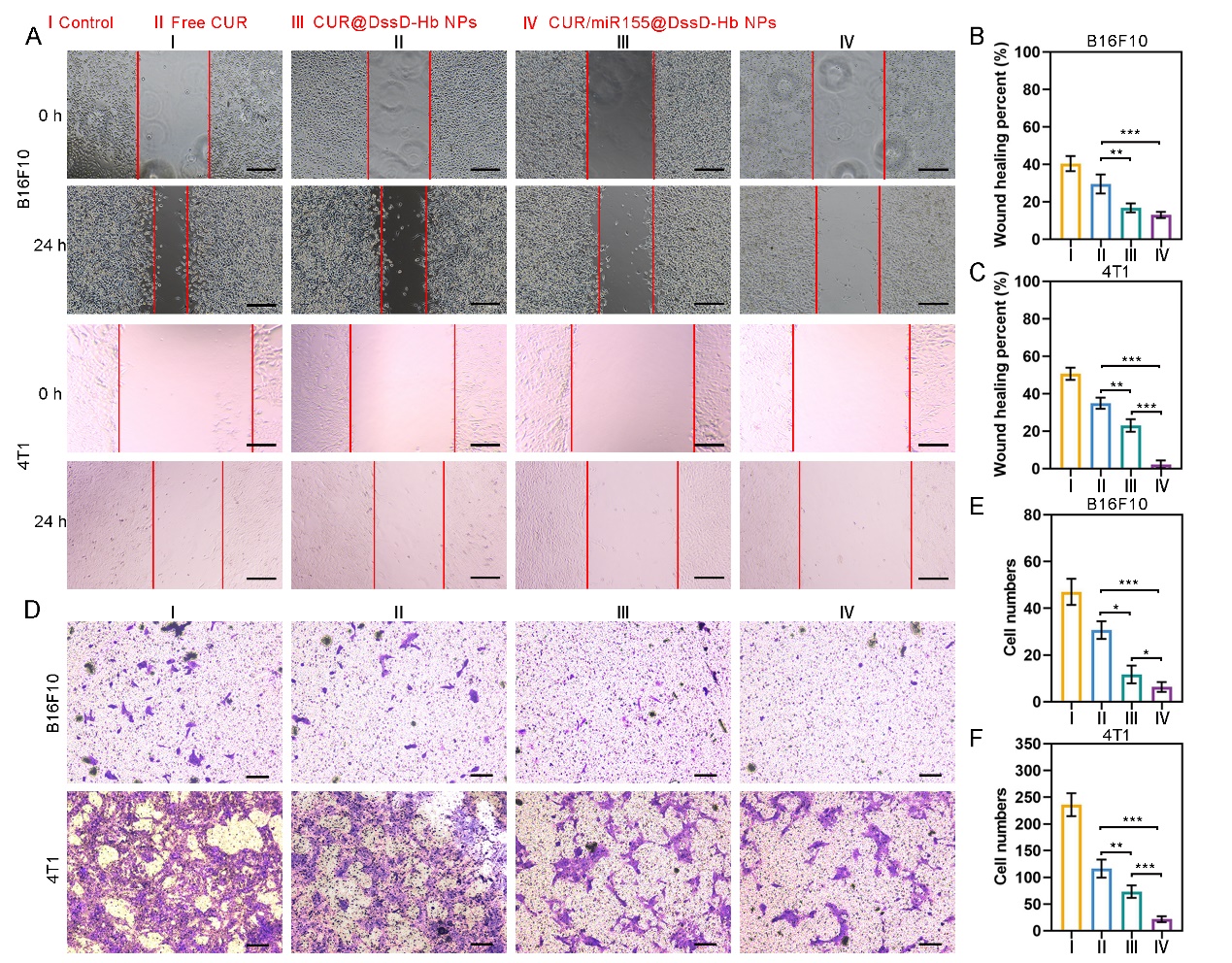


Figure S10 In vitro invasion and migratory assessment. (A) B16F10 and 4T1 cells were wounded, treated with diﬀerent formulations for 48 h, and photographed. The red lines define the areas lacking cells, scale bar = 200 μm. (D) Transwell assays were conducted using B16F10 and 4T1 cells treated with diﬀerent formulations for 48 h, scale bar = 200 μm. Migratory (B and C) and invasion (E and F) analyses of B16F10 and 4T1 cells under diﬀerent formulations. (n = 3, *P < 0.05, **P < 0.01, and ***P < 0.001.)


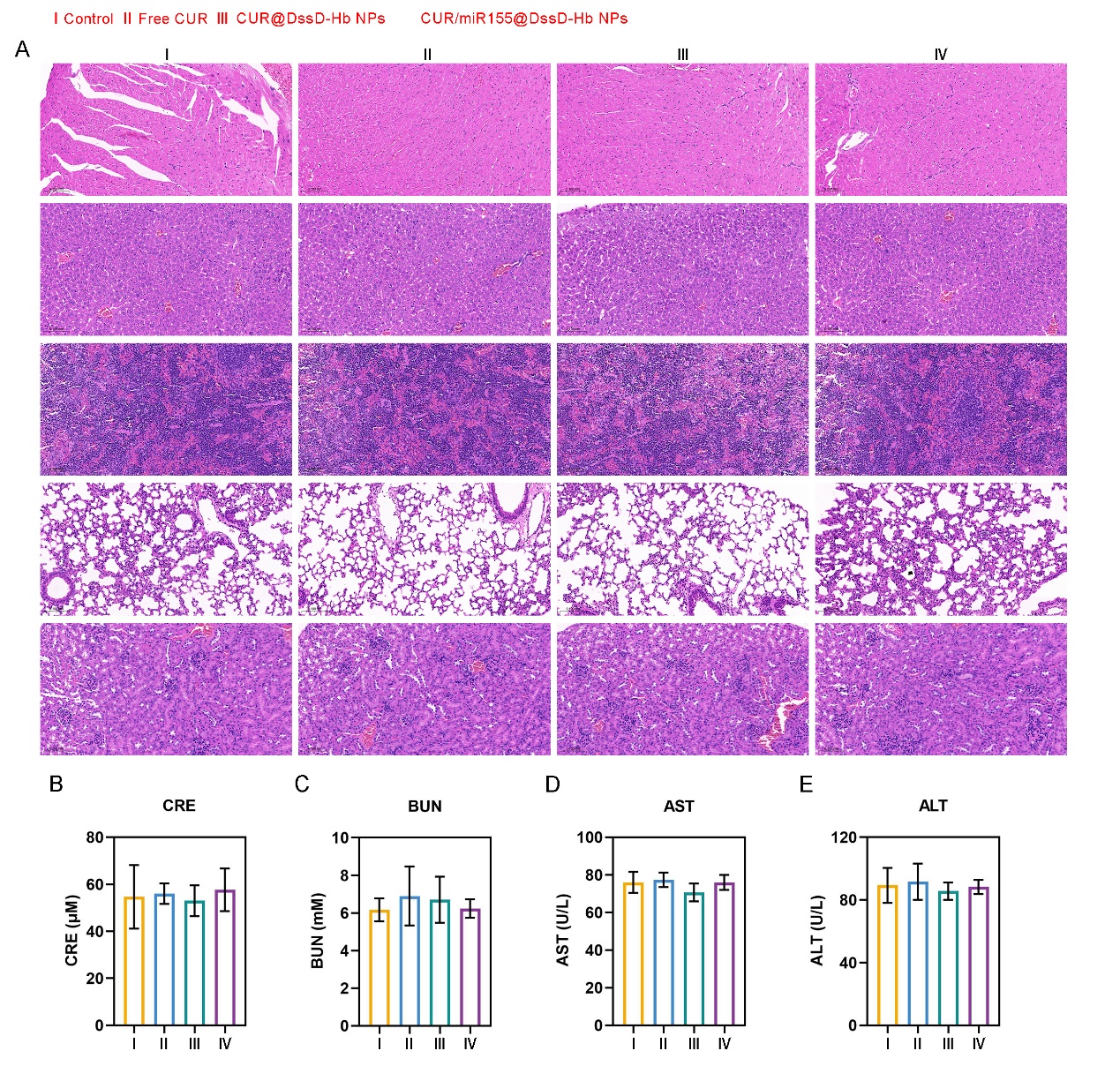


Figure S11 Safety evaluation. (A) H&E staining of major organs obtained from mice receiving diﬀerent treatments, scale bar = 100 μm. (B−E) CRE, BUN, AST, and ALT levels in diﬀerent treatment groups.

**Reference**

1. Zheng, M.; Liu, Y.; Wang, Y.; Zhang, D.; Zou, Y.; Ruan, W.; Yin, J.; Tao, W.; Park, J. B.; Shi, B., ROS-Responsive Polymeric siRNA Nanomedicine Stabilized by Triple Interactions for the Robust Glioblastoma Combinational RNAi Therapy. *Adv Mater* **2019,** *31* (37), e1903277, <https://doi.org/10.1002/adma.201903277>.

2. De Mel, J.; Hossain, M.; Shofolawe-Bakare, O.; Mohammad, S. A.; Rasmussen, E.; Milloy, K.; Shields, M.; Roth, E. W.; Arora, K.; Cueto, R.; Tang, S. C.; Wilson, J. T.; Smith, A. E.; Werfel, T. A., Dual-Responsive Glycopolymers for Intracellular Codelivery of Antigen and Lipophilic Adjuvants. *Mol Pharm* **2022,** *19* (12), 4705-4716, <https://doi.org/10.1021/acs.molpharmaceut.2c00750>.
